# Defining the Functional Role of Na_V_1.7 in Human Nociception

**DOI:** 10.1016/j.neuron.2019.01.047

**Published:** 2019-03-06

**Authors:** Lucy A. McDermott, Greg A. Weir, Andreas C. Themistocleous, Andrew R. Segerdahl, Iulia Blesneac, Georgios Baskozos, Alex J. Clark, Val Millar, Liam J. Peck, Daniel Ebner, Irene Tracey, Jordi Serra, David L. Bennett

**Affiliations:** 1 Nuffield Department of Clinical Neurosciences, University of Oxford, Oxford OX3 9DU, UK; 2 Wellcome Centre for Integrative Neuroimaging, FMRIB, Nuffield Department of Clinical Neurosciences, University of Oxford, Oxford OX3 9DU, UK; 3 Department of Clinical Neurophysiology, King’s College Hospital, London SE5 9RS, UK; 4 Target Discovery Institute, Nuffield Department of Medicine, University of Oxford, Oxford OX3 7FZ, UK

**Keywords:** congenital insensitivity to pain, SCN9A, Na_V_1.7, voltage-gated sodium channel, pain, drug development, induced pluripotent stem cells, CRISPR, nociceptor, microneurography

## Abstract

Loss-of-function mutations in Na_V_1.7 cause congenital insensitivity to pain (CIP); this voltage-gated sodium channel is therefore a key target for analgesic drug development. Utilizing a multi-modal approach, we investigated how Na_V_1.7 mutations lead to human pain insensitivity. Skin biopsy and microneurography revealed an absence of C-fiber nociceptors in CIP patients, reflected in a reduced cortical response to capsaicin on fMRI. Epitope tagging of endogenous Na_V_1.7 revealed the channel to be localized at the soma membrane, axon, axon terminals, and the nodes of Ranvier of induced pluripotent stem cell (iPSC) nociceptors. CIP patient-derived iPSC nociceptors exhibited an inability to properly respond to depolarizing stimuli, demonstrating that Na_V_1.7 is a key regulator of excitability. Using this iPSC nociceptor platform, we found that some Na_V_1.7 blockers undergoing clinical trials lack specificity. CIP, therefore, arises due to a profound loss of functional nociceptors, which is more pronounced than that reported in rodent models, or likely achievable following acute pharmacological blockade.

**Video Abstract:**

## Highlights

• Patients with CIP due to Na_V_1.7 mutations have a functional absence of nociceptors
• Lack of nociceptive drive is reflected in a reduced cortical response to capsaicin
• Patient iPSC nociceptors show reduced excitability, especially to ramp stimuli
• Gene-edited iPSC nociceptors represent a valuable analgesic drug screening platform

## Teaser

Gene mutations associated with painless phenotypes offer great insight for analgesic development. Using a multi-modal approach, McDermott et al. show that mutations in the voltage-gated sodium channel Na_V_1.7 cause congenital insensitivity to pain due to a lack of functional nociceptors.

## Introduction

Bi-allelic inactivating mutations in *SCN9A*, which encodes the voltage-gated sodium channel (VGSC) Na_V_1.7, result in the striking clinical phenotype of congenital insensitivity to pain (CIP) (Cox et al., 2006, Goldberg et al., 2007). These individuals do not perceive pain in response to noxious stimuli (Bennett and Woods, 2014), be it mechanical, thermal, or chemical in form. Conversely, mutations that cause gain of function in Na_V_1.7 have been shown to cause Mendelian human pain disorders such as inherited erythromelalgia (Yang et al., 2004, Dib-Hajj et al., 2005) and paroxysmal extreme pain disorder (Fertleman et al., 2006). Gain-of-function variants were also recently associated with more common acquired pain disorders such as small fiber neuropathy (Faber et al., 2012) and painful diabetic neuropathy (Blesneac et al., 2018).

Na_V_1.7 is highly enriched in nociceptive and sympathetic neurons of the peripheral nervous system (Toledo-Aral et al., 1997). Na_V_1.7 is also expressed in the brain in subcortical structures, including the thalamus, medial amygdala, hypothalamus, and the axons of olfactory epithelium projecting to the olfactory bulb (Kanellopoulos et al., 2018, Branco et al., 2016). Interestingly, the only other clinical feature of bi-allelic loss-of-function (LOF) Na_V_1.7 mutations is anosmia (Weiss et al., 2011). These findings have led to extensive interest in selectively targeting Na_V_1.7 as a means to develop novel analgesics.

Multiple drug development programs have been initiated, including small molecule blockers (Zakrzewska et al., 2017, Cao et al., 2016) and biologic approaches (Lee et al., 2014). Channel selectivity would be highly desirable but is challenging due to the homology of different VGSCs. Non-specific blockers of VGSCs, such as some antiepileptic drugs, are currently used as analgesics; however, the indiscriminate targeting of multiple VGSCs in the heart and/or CNS often lead to significant dose-limiting side effects. A critical barrier to drug development is our current lack of understanding of the mechanisms underlying CIP. We do not yet appreciate the locus of action, and this has important implications for whether novel Na_V_1.7 blockers need to cross the blood-brain barrier. It is also unclear whether there are developmental effects on the structure or function of the sensory nervous system, which would make the CIP phenotype unlikely to be replicated, even by highly potent acute pharmacological blockade.

In rodent, Na_V_1.7 is expressed in the peripheral terminals, axon, soma, and central terminals of sensory neurons (Kanellopoulos et al., 2018, Black et al., 2012). Mutant mouse models in which Na_V_1.7 is globally ablated or conditionally ablated in subsets of sensory neurons have been generated (Nassar et al., 2004, Gingras et al., 2014, Hoffmann et al., 2018). These show reduced (although in some cases not absent) reflex withdrawal responses to a broad range of acute noxious thermal, mechanical, and chemical stimuli on behavioral testing. Interestingly, not all the reduction in pain behavior (i.e., thermal hypoalgesia) appears to be autonomous to sensory neurons but may also involve interaction with the sympathetic nervous system (Minett et al., 2012). Mice lacking Na_V_1.7 in sensory neurons also show reduced hypersensitivity to select neuropathic pain and inflammatory pain models (Minett et al., 2014). Cutaneous innervation by nociceptors is normal in mice with global ablation of Na_V_1.7 (Gingras et al., 2014).

A number of mechanisms, which are not mutually exclusive, have been proposed for this behavioral phenotype. Channel kinetics could position Na_V_1.7 as a threshold channel amplifying sub-threshold depolarizations in nociceptor terminals and by virtue conferring a critical role for Na_V_1.7 in action potential electrogenesis. Na_V_1.7 may also be necessary for action potential propagation along axons and neurotransmitter release at central terminals in an analogous fashion to the olfactory system (Weiss et al., 2011). Finally, Na_V_1.7 may intersect with other signaling systems, such as endogenous opioids, which are upregulated in the absence of Na_V_1.7 and thought to feedback onto dorsal root ganglion (DRG) neurons and/or terminals to suppress excitability (Minett et al., 2015).

There remains an important need to better characterize dysfunction of the somatosensory nervous system in humans with CIP. In rodent DRG neurons, slow membrane depolarization can trigger excitatory ramp currents, driven by Na_V_1.7 activation, which occur as a result of the channel’s slow closed-state inactivation (Cummins et al., 1998). These currents are considered important in amplifying small sub-threshold depolarizing stimuli and thereby increasing the likelihood of an action potential being generated (Dib-Hajj et al., 2013). One recent study using cadaveric human DRG cells from previously healthy donors found a lack of low-threshold excitatory ramp currents (Zhang et al., 2017). The authors concluded that there may be a relative dearth of Na_V_1.7 in human DRG neurons, and although Na_V_1.7 makes a significant contribution to the TTX-sensitive sodium current in rodents, this may not be the case in humans. However, this conclusion is in contrast to another study that found Na_V_1.7 mRNA to be the predominantly expressed VGSC in human DRG tissue (Chang et al., 2018). Zhang et al. (2017) also questioned the selectivity of pharmacological tools to study human Na_V_1.7, but they did not have access to neurons lacking Na_V_1.7 to conclusively determine this.

The techniques available to study the detailed structure and function of the nociceptive system in humans have recently advanced. We have therefore adopted a multi-modal approach including sensory profiling, microneurography, functional brain imaging, and human induced pluripotent stem cell (iPSC) models combined with genome engineering to detail the functional role of Na_V_1.7 in the human nociceptive system.

## Results

### Sensory Profile of CIP Participants

**Figure 1:**
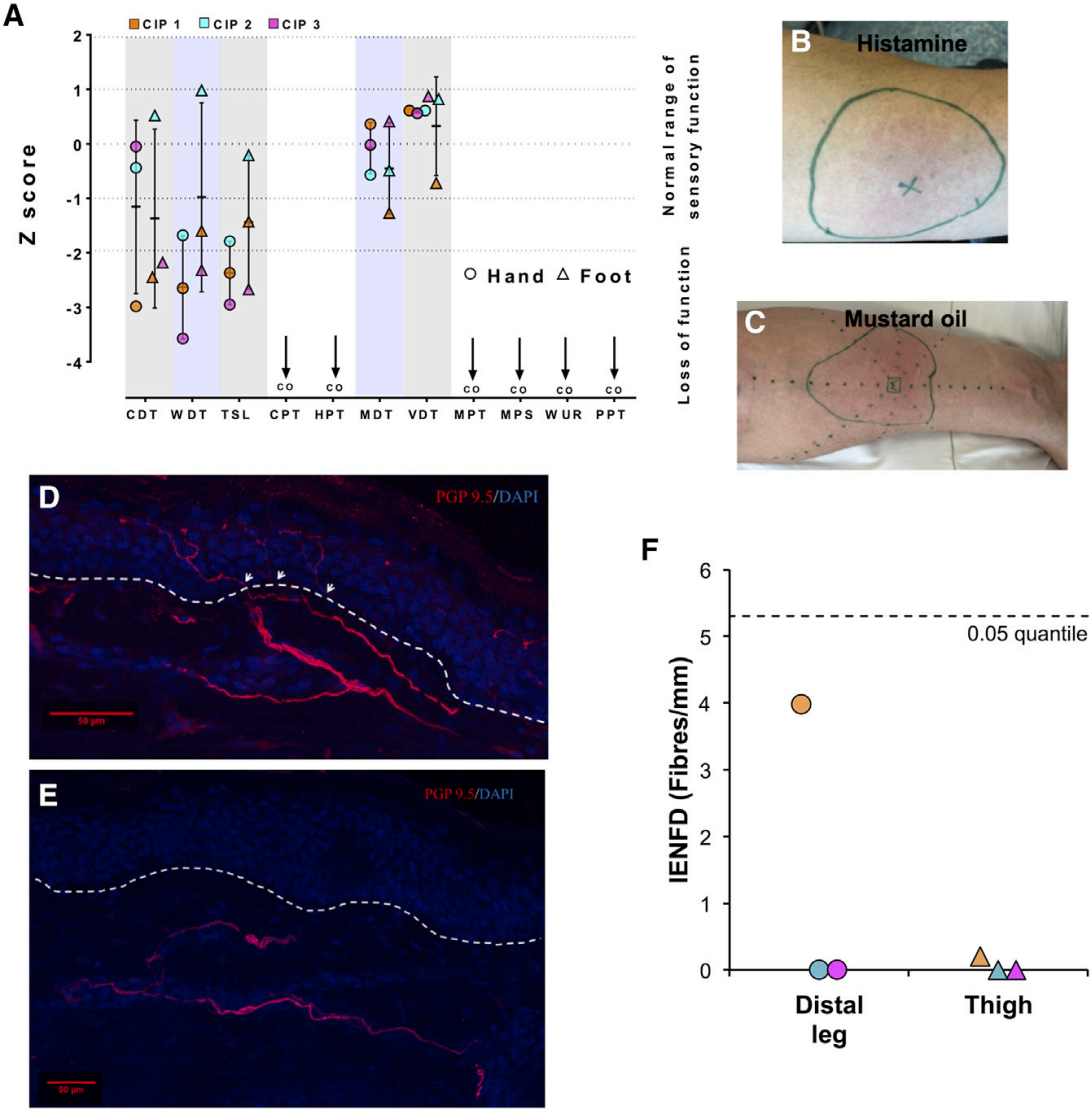
Structure and Function of the Somatosensory Nervous System in CIP Patients (A) Scatterplot of *Z* scores for QST parameters in study participants recorded from the dorsum of the hand and foot. The participants did not feel any pain in response to noxious stimuli and stimuli reached “cut off” (CO on figure). Error bars represent mean ± SD. WDT, warm detection threshold; CDT, cold detection threshold; TSL, thermal sensory limen; CPT, cold pain threshold; HPT, heat pain threshold; MDT, mechanical detection threshold; VDT, vibration detection threshold; MPT, mechanical pain threshold; MPS, mechanical pain sensitivity; WUR, wind-up ratio; PPT, pressure pain threshold. (B) Histamine flare response on the forearm of a CIP participant. “X” marks the area of iontophoresis and the green circle the extent of the flare. (C) Mustard oil flare response on the forearm of a CIP participant. “M” marks the area of mustard oil application and the green circle the extent of the flare response. (D and E) Skin biopsy taken from the lower leg of a healthy control (D) study participant (E) demonstrating the absence of intra-epidermal nerve fibers and the presence of dermal fibers in the CIP study participant. The arrows indicate where the nerve fibers cross between the epidermis and dermis. The dashed line represents the division between the epidermis and dermis. Scale bars represent 50 μm. (F) Quantification of intra-epidermal nerve fibers of skin biopsies taken from the lower leg and proximal thigh from CIP participants. The dashed line represents the lowest 0.05 quantile for published age- and gender-matched normative data. IENFD, intra-epidermal nerve fiber density. See also Figures S1 and S2.

We recruited three CIP participants to assess sensory nerve function (Figure 1; Table 1). All study participants had confirmed compound heterozygous mutations in *SCN9A* that were predicted to cause loss of protein function (Table S1). A summary of their clinical assessment is found in Table 1. All three participants reported multiple painless injuries, including painless fractures, from childhood. On clinical examination, the participants were anosmic and did not feel pinprick as a painful sensation and thus conformed to the typical clinical presentation of *SCN9A*-related CIP (Cox et al., 2006, Weiss et al., 2011). Quantitative sensory testing (QST) confirmed the insensitivity to pain phenotype as neither noxious temperature nor noxious mechanical stimuli were felt as painful (Figure 1A). Cold and warm detection thresholds in both the hand and the foot were reduced when compared to the normative range of the German Neuropathic Pain Consortium, and thermal sensory limen were also impaired (Figure 1A), indicative of thermal hypoesthesia. Mechanical and vibration detection thresholds were normal (Figure 1A). Thus, the QST data showed impairment of small fiber function with preserved large fiber function.

**Table 1:** Summary of Clinical Findings and Special Investigations

	CIP1	CIP2	CIP3
Age	31	34	44
Gender	Male	Male	Female

**History**			

Painless injuries (e.g., fractures, burns)	Y	Y	Y
Congenital onset	Y	Y	Y
Normal cognitive development	Y	Y	Y
Self-mutilation	Y	N	N
Absent visceral pain	Y	Y	Y
Joint replacement	Y	N	Y
Charcot joints	Y	Y	Y
Affected siblings	Y	Y	N

**Examination**			

Anosmia	Y	Y	Y
Normal autonomic function (sweating, bowel function, blood pressure)	Y	Y	Y
Corneal reflex intact	Y	Y	Y
Normal motor examination	Y	Y	Y
Normal deep tendon reflexes	Y	Y	Y
Vibration, mechanical detection, proprioception normal	Y	Y	Y
Pin prick not felt as painful nor sharp	Y	Y	Y
Bedside temperature normal	N	Y	N

**Investigations**			

Histamine flare	Y	Y	Y
Mustard oil flare	Y	Y	Y
Nerve conduction studies normal	N^a^	Y	Y
Threshold tracking normal	Y	Y	Y
fMRI	N	Y	N
Microneurography	Y	Y	Y

**IENFD**			

Proximal thigh (fibers/mm)	0.2	0.00	0.00
Distal leg (fibers/mm) (0.05 quantile; median)	3.98 (5.3; 10.2)	0.00 (5.3; 10.2)	0.00 (5.7; 11.2)

N, no; Y, yes; ND, not done; IENFD, intra-epidermal nerve fiber density.

a Sural amplitudes are reduced

Topical application of algogens or pruritogens to the skin activates cutaneous chemo-sensitive C-fibers and induces release of neuropeptides, causing a neurogenic flare response and resulting in the perception of pain or itch (Groetzner and Weidner, 2010). Histamine (Figures 1B, S1A, and S1B) and mustard oil (Figures 1C, S1C, and S1D) application to the volar surface of the forearm elicited flare responses in all CIP participants. The participants did not report pain or pruritus. This is in contrast to a cohort of healthy controls (n = 10) who unanimously reported a painful “stinging” and “burning” sensation when mustard oil was applied, with a mean maximal visual analog scale (VAS) pain score (95% confidence interval [CI]) of 3.5 (1.8:5.2) (p = 0.03, CIP VAS versus healthy control, Student’s unpaired t test). The histamine flare was blocked when local anesthetic was infiltrated before iontophoresis of the histamine (Figure S1E), indicating that it was neurally mediated. The ability to generate a flare response in CIP participants suggests that at least some cutaneous nerve fibers responsive to noxious stimuli can generate short-range action potentials.

Nerve conduction studies from motor and sensory nerves of the lower limbs, except for one participant, all fell within normative reference ranges (Tables 1 and S2). One participant had small-amplitude sural sensory nerve action potentials. The recordings were difficult due the multiple injuries that the participant had previously sustained to his lower limbs. The reduced amplitudes may therefore be due to past trauma. Sensory nerve excitability measurements recorded from the median nerve all fell within the 95% CIs for healthy study participant parameters (Figure S2). Therefore, functional neurophysiological assessment of large fiber function did not reveal any abnormalities. The intra-epidermal nerve fiber density (IENFD) measurements from the lower leg of our study participants were below the 0.05 quantile for published age- and gender-matched normative data in all cases (Figures 1D–1F). We did, however, observe deeper dermal fibers in all participants (Figure 1E). Therefore, IENFD measurements showed evidence of small fiber pathology. In addition, we also found absent or markedly reduced intra-epidermal nerve fibers at the proximal thigh in all participants (Table 1; Figure S1F).

### Patient *SCN9A* Mutations Result in Loss of Channel Function

**Figure 2:**
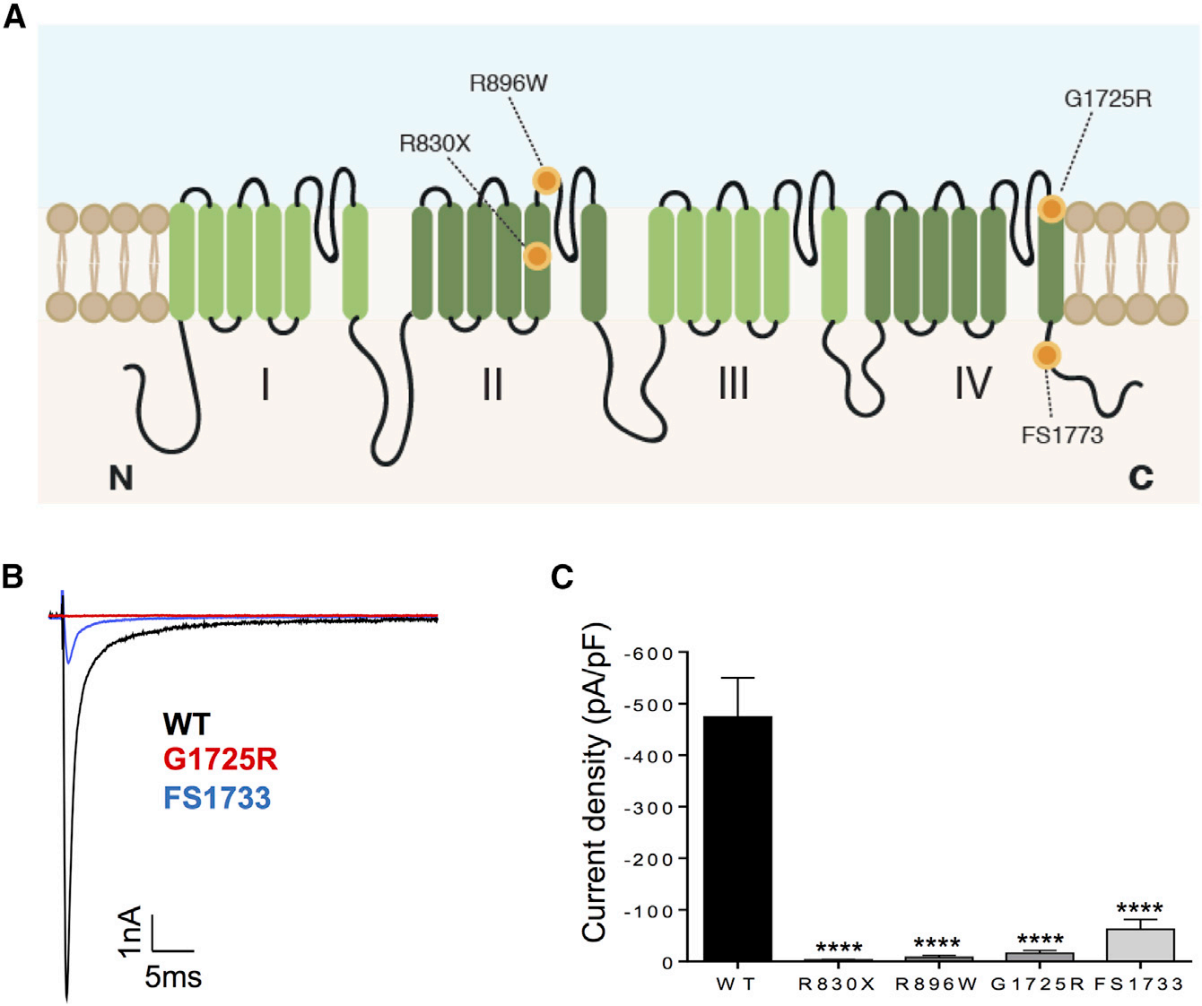
CIP Mutations Result in Loss of Na_V_1.7 Function (A) Schematic of Na_V_1.7 channel topology. CIP mutations are represented with orange dots. (B) Representative currents elicited by a test potential to −10 mV from a holding potential of −100 mV for the wild-type (WT) (black), G1725R (red), or FS1773 (blue) channels. (C) Summarized data for whole-cell current density elicited by a test potential to −10 mV from a holding potential of −100 mV for the WT (−474.2 ± 75 pA/pF, n = 17), R896W (−7.6 ± 3.8 pA/pF, n = 9), R830X (−2.8. ± 0.9 pA/pF, n = 8), G1725R (−15.7 ± 5.4 pA/pF, n = 9), and FS1773 (−62.2 ± 18.8, n = 8). Data are presented as mean ± SEM. For all, ^∗∗∗∗^p ≤ 0.0001 compared with WT. One-way ANOVA followed by Dunn’s multiple comparison test.

Only one mutation (c.2691G > A p.Y897X) in our patient cohort has previously been described and functionally characterized (Cox et al., 2006). We heterologously expressed the novel Na_V_1.7 variants (Figure 2A) in HEK293T cells and used whole-cell patch-clamp recordings to evaluate their impact on channel biophysics. Representative whole-cell voltage-clamp currents from transfected cells are shown in Figure 2B. All the mutations drastically reduced Na_V_1.7 current. FS1773 mutation resulted in an 8-fold reduction in the current density of the channel (Figures 2B and 2C). The R896W, R830X, and G1725R showed a profound loss of function with negligible current densities (Figure 2C). All CIP mutations therefore caused a significant loss of conductance compared to control, consistent with almost complete loss of channel function.

### C-Fiber Nociceptors Are Not Detected by Microneurography

**Figure 3:**
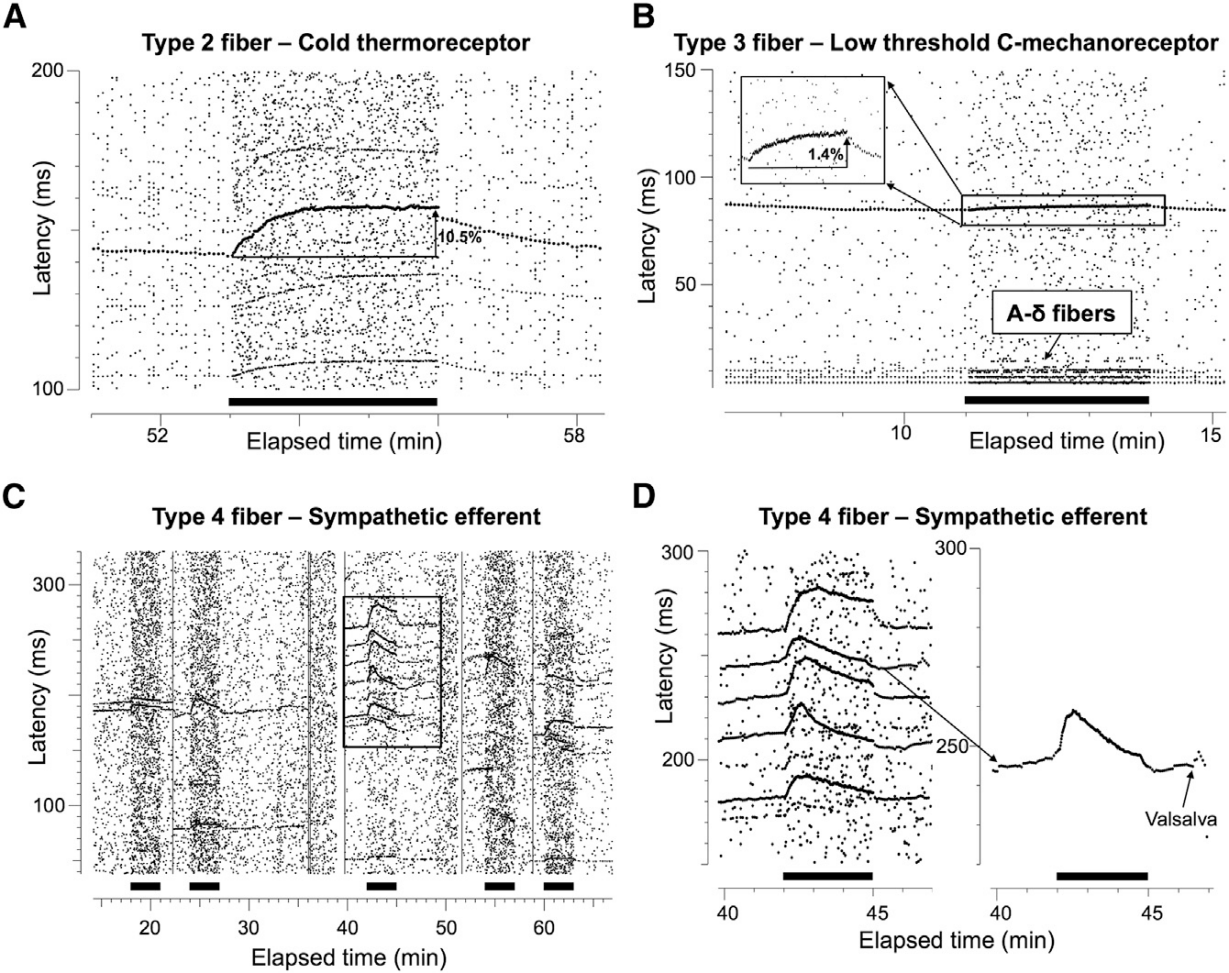
Microneurographic Raster Plots Illustrating Absence of ADS Profiles Compatible with C-Nociceptors (A–D) Several characteristic profiles of activity-dependent slowing were identified in all three patients compatible with cold thermoreceptors (A), low threshold C-mechanoreceptors (B, longer latency), Aδ fibers (B, shorter latencies), and several sympathetic units (C and D). However, no ADS profiles characteristic of C-nociceptors could be identified. (C) 5 different intraneural recording sites separated by straight vertical lines in which only profiles of ADS compatible with sympathetic units could be recorded with an initial slowing followed by relative speeding up of conduction velocity during a 2 Hz stimulation period for 3 min (marked by filled bars). (D) Magnified view of the square of the third intraneural recording site in (C). Right shows an enlarged view of a sympathetic unit at initial latency of 240 ms responding to a Valsalva maneuver with a burst of activity. Nomenclature of units follows that of Serra et al., 1999, Serra et al., 2004.

A total of 38 C-fibers were recorded and analyzed from three of the subjects (14 from 4 intraneural sites in patient CIP1, 7 from 3 intraneural sites in patient CIP2, and 17 from 6 intraneural sites in patient CIP3). None of these recordings identified fibers with properties consistent with C-nociceptors. The frequency of different profiles of activity-dependent slowing (ADS) of conduction velocity was: type 1 (C-nociceptors) 0 (0%), type 2 (thermoreceptors) 10/38 (26.3%), type 3 (low-threshold C-mechanoreceptors) 5/38 (13.2%), and type 4 (sympathetic efferent) 23/38 (60.5%) (Figure 3). All intraneural sites showed ADS profiles with “plateau” units during 2 Hz stimulation (see for comparison Figure 3 in Serra et al., 1999, Figure 1 in Serra et al., 2004, or Figure 1 in Campero et al., 2004). This frequency of sensory afferent types was significantly different to normative data from healthy patients (Serra et al., 1999, Campero et al., 2004), owing to the lack of type 1 fibers (p < 0.001, Fisher’s exact test). Therefore, there was a striking lack of ADS of conduction velocity profiles compatible with peripheral C-nociceptors. There were no differences with historical ADS of conduction velocity data for these three C-fiber types (Table S3).

### Brain Activity Changes in Response to Capsaicin Are Reduced in a CIP Patient

Despite the stark clinical phenotype of CIP, a previous study failed to find differences in brain activity of CIP patients compared to healthy controls in response to acute noxious mechanical stimuli (Salomons et al., 2016). However, the method used for comparison in this study has been criticized, making the conclusions from the original study less clear (Büchel et al., 2016).

**Figure 4:**
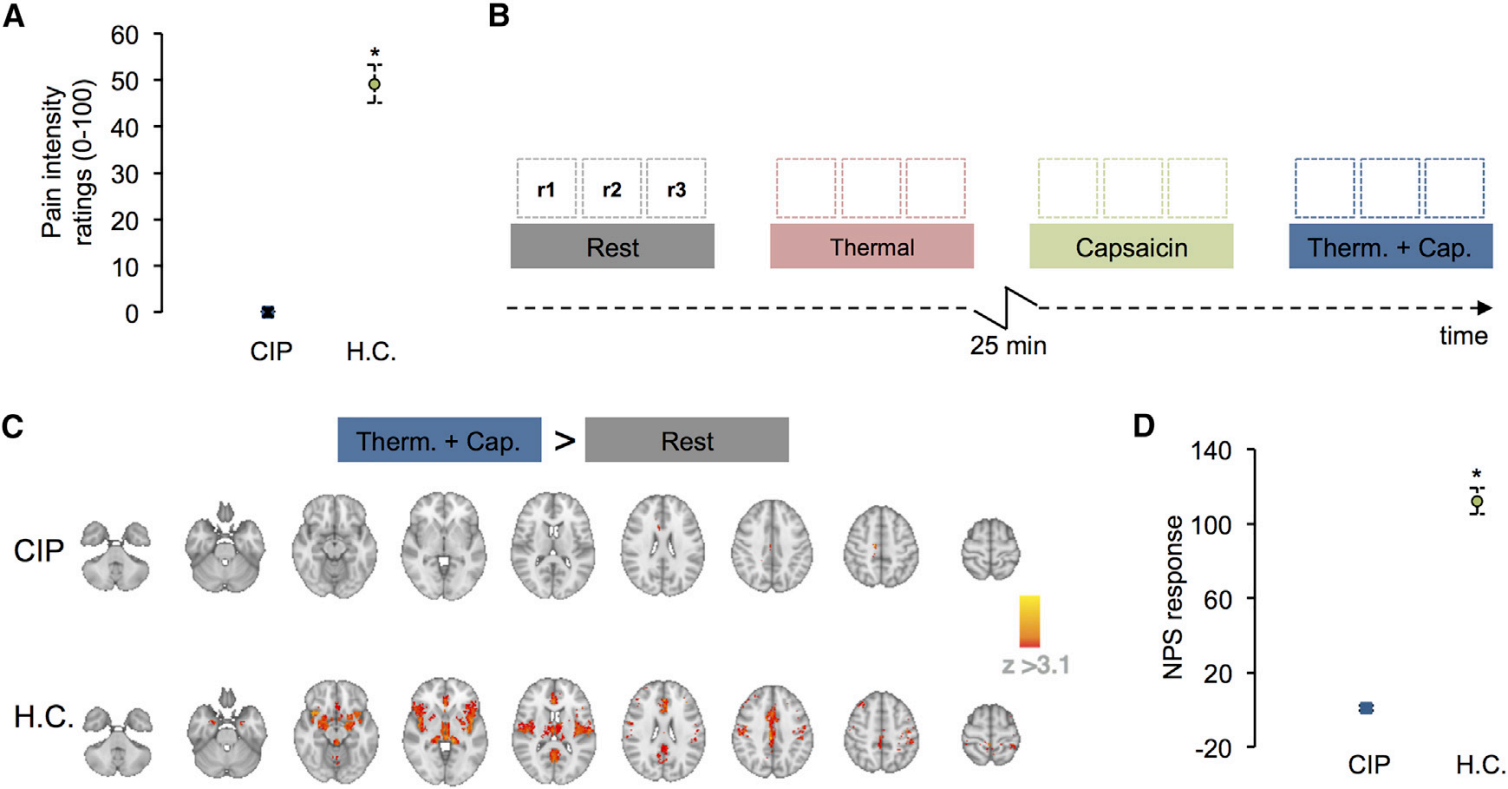
Reduced Cortical Response in CIP Patient to Capsaicin Challenge (A) The comparison of the group mean verbal pain intensity ratings collected during the “thermal + capsaicin” condition between the CIP and healthy controls (HC) (Mann-Whitney U test, ^∗^p < 0.001). For HC, the error bars represent the SEM for the group (n = 12). Here, each healthy control participant was scanned once. During this scan, a single trial testing the effects of each experimental condition once was observed for that subject. Multiple-repeat trials were not collected on the HC cohort. For the single CIP patient, the error bars represent the SEM across repeated trials for that patient. The CIP patient was scanned twice. In each CIP scan session, three repeat trials were collected for each experimental condition. A total of six trials were included in the analysis of the CIP patient. (B) A schematic of the experimental paradigm used to image CIP patient. The schematic represents a single scan session. The four experimental conditions: Rest, Thermal, Capsaicin, and Thermal + Capsaicin (“Therm.+Cap.”) are displayed as colored boxes. Three 5-min scan runs (i.e., r1, r2, r3) were collected for each condition. The 25-min capsaicin onset phase is displayed as an inverted “z.” No FMRI data were collected here. (C) The mean change in CBF elicited by the contrast of “thermal + capsaicin versus rest” for CIP (top row) and healthy controls (HC) (bottom row) (mixed effects; z > 3.1, p < 0.05). Regions showing an increase in CBF during “thermal + capsaicin > rest” are displayed in red. (D) Comparison of the group mean neurological pain signature (NPS) response values observed from the “thermal + capsaicin > rest” contrast images at the subject level (Mann-Whitney U test, ^∗^p < 0.001). Data from repeated trials were processed as in (A).

We sought to investigate tonic responses to an algogen (capsaicin) using arterial spin labeling in one CIP participant (CIP2) who was eligible for functional brain imaging. This approach removes the confound of mechanical stimulation and avoids the analysis and interpretative problems pointed out previously (Büchel et al., 2016). Treatment of the CIP participant’s skin with a 1% topical capsaicin cream failed to elicit similar responses to what is commonly reported in a healthy control (HC) population. The participant reported no pain and no unpleasantness associated with any of the experimental conditions tested (NRS = 0). This was in contrast to the HC group, which found the experience of capsaicin and thermal stimulation intensely painful (NRS = 49.997 ± 3.525) (Figure 4A; ^∗^p < 0.001 Mann-Whitney U test). In CIP, the brain response to “thermal + capsaicin” versus “rest” was the only contrast that showed significant changes in cerebral blood flow (CBF). Increased CBF was observed in the primary somatosensory cortex (SI), the dorsal anterior cingulate, and the posterior cingulate (mixed effects; z > 3.1, p < 0.05) (Figure 4C). In healthy controls, significant increases in CBF were observed in the primary and secondary somatosensory cortices, dorsal lateral prefrontal cortex, insula (anterior, mid, and posterior), anterior cingulate, putamen, nucleus accumbens, periaqueductal gray, and the cerebellum (mixed effects; z > 3.1, p < 0.05) (Figure 4C). To confirm that the changes observed during the capsaicin-evoked thermal condition are related to distinct perceptual experiences in CIP versus HC, we applied the neurological pain signature (NPS) to these data. The NPS is a weighted multivariate brain activation map that is strongly correlated with experimental physical pain reports and can be applied to imaging data to predict the severity of pain that was likely experienced during a scan (Wager et al., 2013). For each subject, the NPS expression was calculated by taking the dot product of the NPS and the contrast image for “thermal + capsaicin > rest” condition. For CIP, the NPS expression was generated from the mean of six repeated trials of the “thermal + capsaicin > rest” conditions. For HC, the NPS was derived from the mean across 12 subjects scanned once. A comparison of the NPS response values observed was significantly greater in HC compared to CIP (^∗^p < 0.001 Mann-Whitney U test) (Figure 4D). In effect, there was no overlap with the NPS of brain activity from the CIP patient in response to capsaicin-induced tonic heat hyperalgesia, whereas there was overlap with the NPS of brain activity from HC.

### Na_V_1.7 Is Robustly Expressed in iPSC Nociceptors and Trafficked to Specific Neuronal Compartments

**Figure 5:**
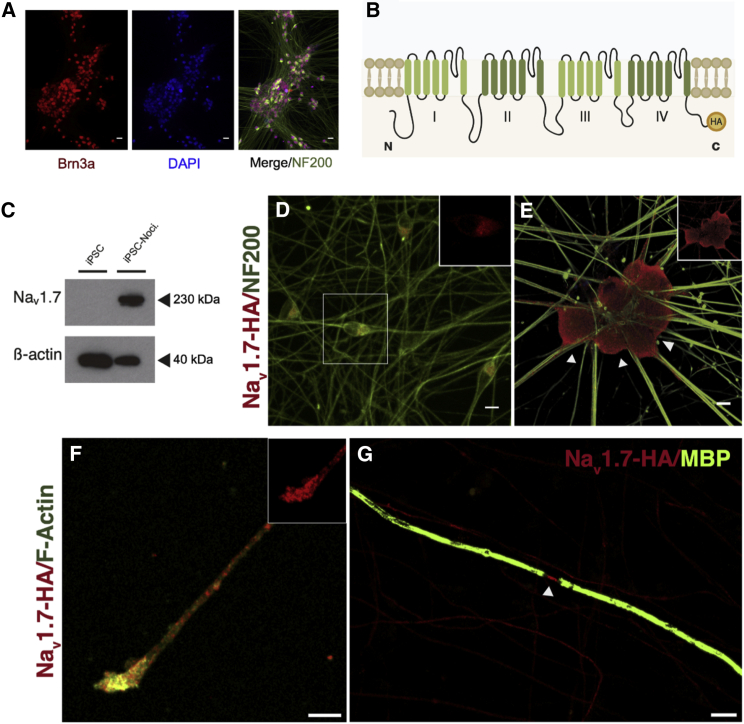
Genome Tagging of Na_V_1.7 Identifies Precise Subcellular Localization (A) Representative split channel image of iPSC nociceptors following differentiation, Brn3a (sensory marker, red), DAPI (nuclei, blue), and NF200 (neurofilament, green). (B) Na_V_1.7 membrane topology, the location of the C-terminal HA epitope tag is indicated in yellow. (C) Representative immunoblot of Na_V_1.7 protein expression in SCN9A-HA iPSC and 60 DIV SCN9A-HA iPSC nociceptors; loading control β-actin. (D and E) Immunocytological co-staining of 25 DIV (D) and 60 DIV (E) iPSC nociceptors, NF200 (green), Na_V_1.7-HA (red), inset image depicts separated channel Na_V_1.7-HA stain. Arrows in (E) indicate marked cell surface expression. (F) Representative image showing Na_V_1.7 expression in the axonal terminals of mature cultures, F-Actin (green), Na_V_1.7-HA (red), inset; separated channel image of Na_V_1.7-HA stain. (G) Na_V_1.7 localized to the node of Ranvier (arrow) in a myelinated co-culture, myelin basic protein (green), Na_V_1.7-HA (red). Scale bars, 25 μm (A) and 10 μm (D–G). See also Figures S3 and S4.

The directed differentiation of human iPSCs to nociceptors enables functional modeling of CIP pathogenesis *in vitro* and should provide significant insight into the role of Na_V_1.7 in nociceptor physiology. We used the differentiation protocol described by Chambers et al. (2012) to generate highly pure cultures of neurons that express the sensory neuron marker Brn3a (Figure 5A). Mature iPSC nociceptors are molecularly comparable to human sensory neurons, capable of responding to noxious stimuli, and exhibit mature electrophysiological characteristics (Young et al., 2014, Chambers et al., 2012, Weir et al., 2017). We further confirmed the molecular profile of our iPSC nociceptors by performing RNA sequencing (RNA-seq). Principal-component analysis demonstrated good clustering of iPSC nociceptors with previously published studies (Schwartzentruber et al., 2018) and with hDRG data tissue (Ray et al., 2018) (Figure S3A). iPSC nociceptors had high DRG neuronal signature (compiled from enriched genes in hDRG tissue data) relative to other tissues (Figure S3B). *SCN9A* expression was found to be high and to a similar level to hDRG tissue (Figure S3C). We then sought to examine the distribution of Na_V_1.7 protein in iPSC nociceptors. Commercially available antibodies targeting the human channel are poor, hampering efforts to localize Na_V_1.7 protein. We therefore decided to tag endogenous Na_V_1.7 using CRISPR-Cas9-mediated genome editing (Cong et al., 2013). The highly antigenic epitope tag hemagglutinin (HA) was knocked in frame to the C terminus of one *SCN9A* allele (Figures 5B and S4), labeling all known functional coding Na_V_1.7 transcripts.

We examined temporal changes in expression of Na_V_1.7 following differentiation and found marked differences in protein expression and localization through maturation. Na_V_1.7 was absent in iPSC cultures but highly enriched in matured iPSC nociceptors (Figure 5C). A low level of neuronal expression was observed at 25 days *in vitro* (DIV); protein was restricted to the cell body and concentrated in a peri-nuclear hemi-ring consistent with endoplasmic reticulum (ER) retention (Figure 5D). In contrast, in mature cultures (60 DIV), we observed a striking enrichment in cell surface expression and staining that spanned the entire axonal trajectory (Figure 5E). Na_V_1.7 has previously been reported to localize to axon terminals in rodent DRG neurons (Black et al., 2012). Consistent with this finding, staining revealed enrichment of Na_V_1.7 in terminal structures of our iPSC nociceptors (Figure 5F). We have previously established that iPSC nociceptors can be efficiently myelinated *in vitro* when cultured with rat Schwann cells (Clark et al., 2017). Having revealed robust expression in unmyelinated human nociceptors, we next examined Na_V_1.7 localization in myelinated co-cultures. Na_V_1.7 could be seen localized to >90% of nodes of Ranvier in myelinated axons—demarcated and flanked by MBP expression (Figure 5G). In immature nodes present within the culture, channel distribution was concentrated but notably elongated, consistent with the role of the paranodes in concentrating VGSC localization at nodes (Amor et al., 2017) (Figure S5A). *In vivo*, C-fibers associate with non-myelinating Schwann cells to form Remak bundles, so we therefore also examined the influence of Schwann cells on Na_V_1.7 localization in co-cultures in which myelination was not induced. Axons juxtaposed to aligned non-myelinating Schwann cells exhibited Na_V_1.7 localization along the length of the neurite, similar to those cultured in the absence of Schwann cells (Figure S5B). Together, these results demonstrate that Na_V_1.7 is highly expressed in iPSC nociceptors and is trafficked appropriately to polarized neuronal compartments.

### Na_V_1.7 Modulates Excitability of iPSC Nociceptors

**Figure 6:**
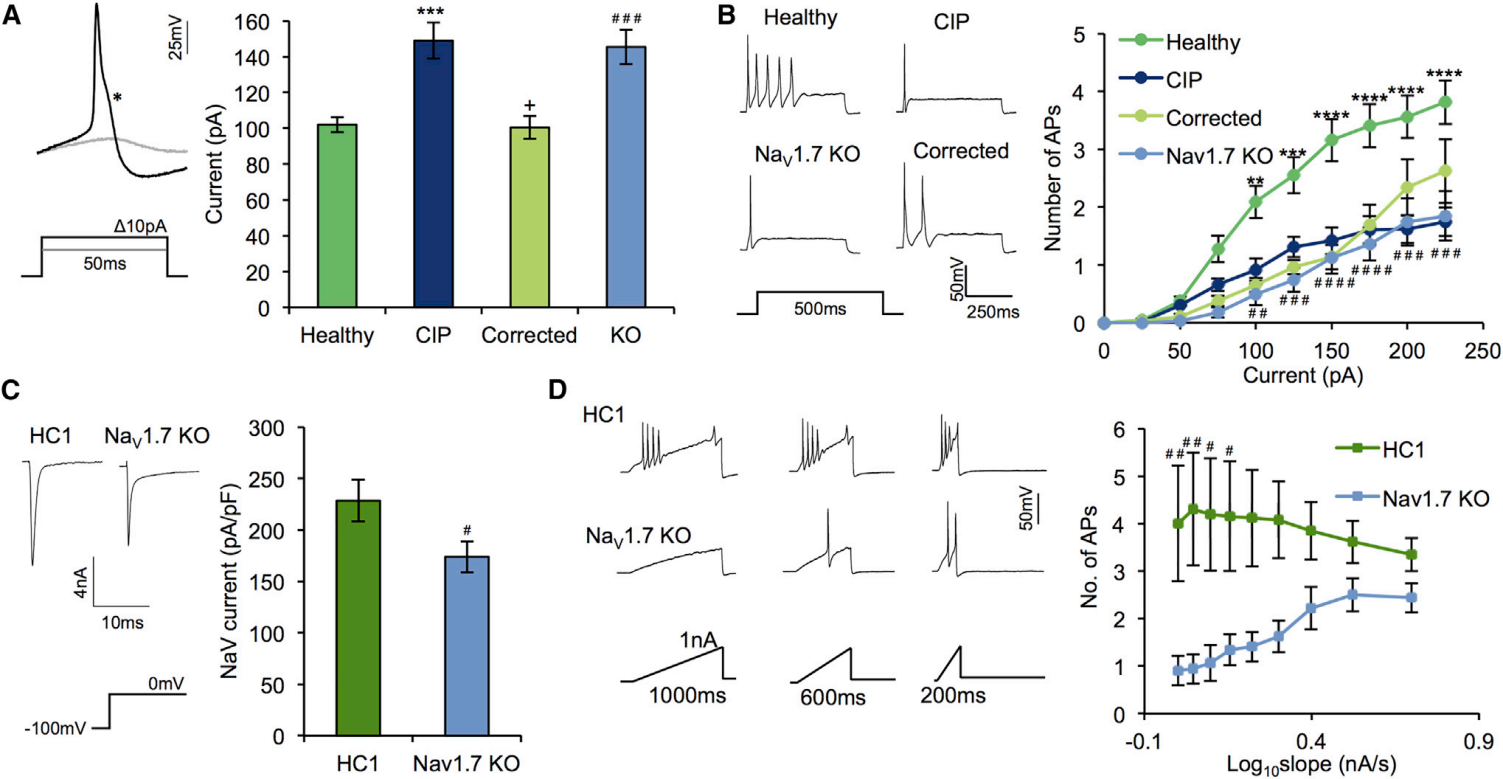
Na_V_1.7 Modulates Somal Excitability of Human Nociceptors (A) Left: representative image showing action potential firing of a DIV 60 iPSC nociceptor to incremental current injections. Note the inflection on the falling phase of the action potential. Right: rheobase derived from pooled data of healthy (119 cells), CIP (85 cells), corrected (33 cells), and Na_V_1.7 KO (45 cells) groups. Kruskal-Wallis followed by post hoc Dunn’s test was used for all comparisons. (B) Supra-threshold firing in response to prolonged depolarization current injection. Left: representative firing from a cell from each group. Right: pooled data from the same cells as (A). Two-way ANOVA followed by post hoc Sidak’s multiple comparison test. (C) Left: representative trace of voltage-gated sodium (Na_V_) current induced by step depolarization to 0mV from a holding potential of −100 mV. Right: quantification of maximal NaV current comparing HC1 (n = 31) and Na_V_1.7 KO (n = 27) groups. Student’s unpaired t test. (D) Left: representative traces of firing in response to 1 nA current injection given over 100–1000 ms in 100 ms increments. Right: quantification of firing across different slopes of current injection. Two-way ANOVA followed by post hoc Sidak’s multiple comparison test. (HC1, 44 cells, and Na_V_1.7 KO, 54 cells). See also Figure S6. All data represent mean ± SEM pooled from at least independent differentiations. ^∗∗^p < 0.01, ^∗∗∗^p < 0.001, ^∗∗∗∗^p < 0.01 healthy versus CIP. ^#^p < 0.05, ^##^p < 0.01, ^###^p < 0.001, ^####^p < 0.0001 Na_V_1.7 KO versus HC1 (parent clone). ^+^p < 0.05 corrected versus cCIP1.2 (parent clone).

To investigate the contribution of Na_V_1.7 to nociceptor function, we derived iPSC nociceptors from healthy and CIP donors. Two clones were generated from patient CIP1 (clones cCIP1.1 and cCIP1.2) and one clone from patient CIP2 (clone cCIP2). Three clones generated from healthy control donors (HC1, HC2, and HC3) were used as comparators. Human and rodent nociceptors exhibit an inflection on the falling phase of the action potential (Davidson et al., 2014, Ritter and Mendell, 1992); thus, to purify recordings for mature iPSC nociceptors, we only included recordings from neurons that demonstrated this feature (Figure 6A). Healthy and CIP iPSC nociceptors did not differ in their resting state biophysical properties, including membrane potential (Table S4). When firing properties were assessed, CIP iPSC nociceptors were found to be less excitable than healthy controls. CIP iPSC nociceptors required increased current stimuli to generate an action potential (healthy control 102.1 ± 4.2 pA versus CIP 149.0 ± 10.0 pA, p < 0.001) (Figures 6A and S6A) and fired fewer action potentials in response to prolonged (500 ms) supra-threshold depolarization compared to healthy controls (Figures 6B and S6B). To confirm that the observed hypoexcitability was due to a loss of Na_V_1.7 function, we used CRISPR Cas9 to correct one *SCN9A* allele of clone CIP1.2, reversing the c2488c > t mutation to the wild-type sequence (Figure S4). The corrected clone (corrected) demonstrated a reduced rheobase compared to CIP1.2 (CIP1.2 142.7 ± 13.7 pA versus Corrected 97.1 ± 5.5 pA, p < 0.05) (Figures 6A and S6A) that was similar to healthy controls (Figure 6A). The patient phenotype of reduced firing in response to prolonged supra-threshold stimuli was not reversed by genetic correction (Figures 6B and S6B). These results are consistent with the recessive nature of CIP but suggest that to properly sustain repetitive firing, neurons require two functional copies of *SCN9A*.

To study the function of Na_V_1.7 independent of inter-patient genetic variability, we introduced a homozygous *SCN9A* frameshift mutation (N842X) into a healthy control iPSC line (HC1), generating a Na_V_1.7 knockout (Na_V_1.7 KO) (Figure S4B). The *de novo* mutation produces a premature stop codon in domain II of the channel, leading to nonsense-mediated decay of the *SCN9A* mRNA transcript (Figure S4C). Na_V_1.7 KO iPSC nociceptors recapitulated the excitability changes observed in CIP lines, including an increased rheobase (HC1 101.6 ± 5.3 pA versus Na_V_1.7 KO 145.5 ± 9.7 pA, p < 0.001) (Figures 6A and S6A) and a decreased propensity to fire repetitively to supra-threshold stimulation (Figures 6B and S6B). A recent report proposed that loss of Na_V_1.7 leads to CIP at least partially through upregulation of endogenous opioids, in particular proenkephalin (PENK) (Minett et al., 2015). In the same study, naloxone (an opioid antagonist) was shown to reduce analgesia in one CIP patient. We tested the efficacy of naloxone treatment to normalize hypoexcitability of Na_V_1.7 KO and found no effect on excitability measures of Na_V_1.7 KO neurons (Figures S5E–S5G), demonstrating that opioid upregulation was not driving the cellular phenotype that we observed. Consistent with these results, we failed to detect PENK mRNA in healthy control or Na_V_1.7 KO iPSC nociceptors by qPCR (data not shown) and did not detect meaningful expression (TPM < 1) in healthy control iPSC nociceptors by RNA-seq analysis. Using whole-cell voltage-clamp recordings, we found that the peak voltage-gated sodium current was reduced by 23.89% in Na_V_1.7 KO neurons compared to control (Figure 6C). This finding is in line with studies in mouse assessing the contribution of mNa_V_1.7 to voltage-gated sodium currents in small-diameter nociceptors (Gingras et al., 2014, Minett et al., 2012). Stimulation of nociceptor peripheral terminals likely results in generator potentials, which slowly depolarize the membrane potential (Waxman 2006). Na_V_1.7 displays slow closed-state inactivation, which enables the channel to open in response to ramp depolarization and initiate action potential generation (Cummins et al., 1998). To test the hypothesis that Na_V_1.7 has a role in enabling neurons to respond to slow depolarization, we injected ramps of supra-threshold current with increasing gradients to mimic generator potentials. Na_V_1.7 KO iPSC nociceptors were markedly less responsive to ramp depolarization, especially to slower stimuli (Figure 6D). Supporting this finding, CIP iPSC nociceptors were also less responsive compared to healthy control (Figures S6C and S6D). These data confirm the role of Na_V_1.7 in setting the excitability state of iPSC nociceptors. Given the finding of reduced IENFD in CIP patients and a previous report linking Na_V_1.7 gain-of-function mutations to altered neurite outgrowth of sensory neurons *in vitro* (Persson et al., 2013), we sought to test whether iPSC nociceptors lacking Na_V_1.7 had a defect in neurite outgrowth. Mature iPSC nociceptors develop substantial projections during their time in culture (Figure S7A). We quantified the area covered by these projections and found no difference between Na_V_1.7 KO iPSC nociceptors and an isogenic control line (HC1) (Figure S7B). Analogous to rodent DRG, when iPSC nociceptors are dissociated, they regenerate neurites within hours (Figure S7C). A similar proportion of nociceptors lacking Na_V_1.7 (CIP and Na_V_1.7 KO) generated neurite projections 12 h following dissociation, compared to healthy control iPSC nociceptors (χ^2^ (2) = 3.425, p = 0.18) (Figure S7D). Of neurons that established neurites, the average length was also not different between the groups (Figure S7E). These results suggest that *in vitro*, loss of Na_V_1.7 does not impact on intrinsic neurite outgrowth.

### Na_V_1.7 KO as a Platform for Analgesic Drug Screening

**Figure 7:**
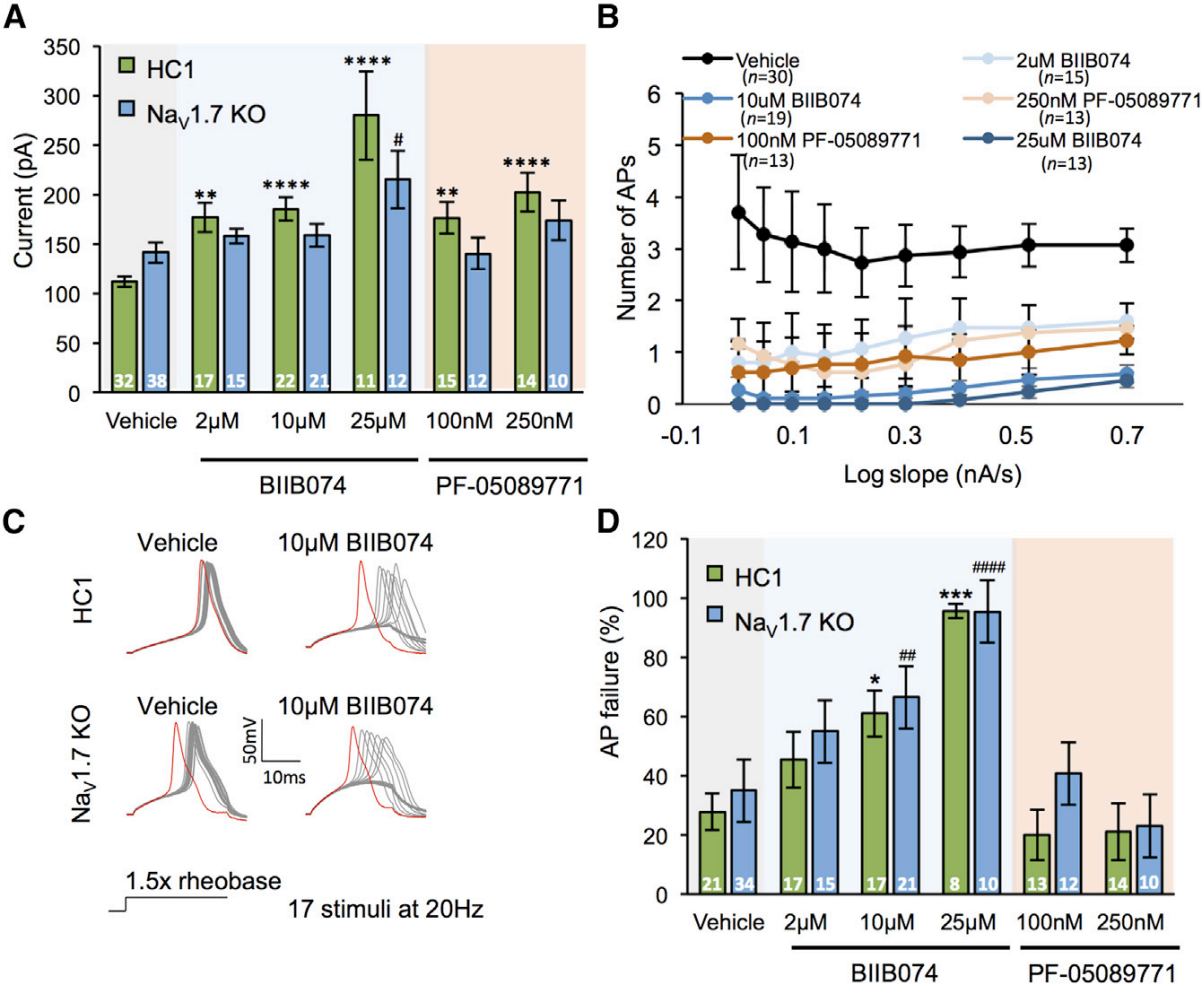
Na_V_1.7 Selectivity of Clinical Compounds (A) Rheobase of HC1 and Na_V_1.7 KO neurons treated with vehicle (0.01% DMSO) or varying concentrations of the Na_V_1.7 blockers, BIIB074, and PF-05089771. Number of recordings are denoted in the bars, Kruskal-Wallis followed by Dunn’s multiple comparison test. (B) Response of HC1 neurons to ramp depolarization with and without drug treatment. (C) Representative traces of action potential firing in response to multiple depolarizing current pulses given at 20 Hz. Red traces signify the action potential generated in response to the 1^st^ current step. (D) Quantification of action potential failures in response to multiple depolarizing current pulses given at 20 Hz following vehicle or drug treatment. Number of recordings are denoted in the bars. Kruskal-Wallis followed by Dunn’s multiple comparison test. Results pooled from at least three independent experiments and represent mean ± SEM. ^∗^p < 0.05, ^∗∗^p < 0.01, ^∗∗∗^p < 0.001, ^∗∗∗∗^p < 0.0001 HC1 treatment versus vehicle control. ^#^p < 0.05, ^##^p < 0.01, ^####^p < 0.0001 Na_V_1.7 KO treatment versus vehicle control.

Due to the genetic linkage between *SCN9A* LOF mutations and CIP, developing specific inhibitors of Na_V_1.7 has become a major goal of pharmaceutical companies seeking to generate novel analgesics (Yekkirala et al., 2017). We reasoned that our iPSC Na_V_1.7 KO lines would act as a platform to validate the specificity of selective inhibitors. BII074 is a state-dependent blocker that is considered selective for Na_V_1.7 (Zakrzewska et al., 2017). BII074 was recently shown to be safe and to have some efficacy in treating trigeminal neuralgia patients (Zakrzewska et al., 2017). Clinically relevant concentrations of BII074 dose-dependently increased the rheobase and reduced the response to slow ramp depolarization of control (HC1) iPSC nociceptors, consistent with block of Na_V_1.7 (Figures 7A and 7B). At concentrations observed in treated patient plasma (2–10 μM) (Zakrzewska et al., 2017), BII074 had no effect on rheobase of Na_V_1.7 KO neurons (Figure 7A). However, selectivity was not observed at a moderately increased concentration (25 μM), indicating that at these levels BII074 acts to modulate non-Na_V_1.7 ion channels to influence excitability (Figure 7A). *In vitro*, BIIB074 preferentially inhibits firing in response to high-frequency stimulation (Zakrzewska et al., 2017), which may occur during paroxysmal pain attacks (as are frequently reported in trigeminal neuralgia). To test high-frequency block, we injected current steps of 150% rheobase magnitude at 20 Hz and measured the ability of neurons to faithfully fire action potentials in response to depolarization. Na_V_1.7 demonstrates slow recovery from inactivation and therefore is not likely to contribute to firing at this frequency (Herzog et al., 2003, Dib-Hajj et al., 2013). BII074 dose-dependently induced high-frequency firing block in control but also Na_V_1.7 KO neurons (Figures 7C and 7D). This result suggests that BII074 inhibits neuronal firing in the absence of Na_V_1.7, and at this concentration, the drug is acting on other targets.

PF-05089771 is a potent arylsulfonamide Na_V_1.7 inhibitor (Alexandrou et al., 2016) undergoing clinical trials for painful diabetic neuropathy (McDonnell et al., 2018) and inherited erythromelalgia (Cao et al., 2016). At concentrations equivalent to the free plasma concentration of PF-05089771 measured in treated patients (Cao et al., 2016), PF-05089771 increased the rheobase of control, but not Na_V_1.7 KO, neurons (Figure 7A). PF-05089771 did not affect high-frequency firing of either group of neurons at any dose trialed (Figures 7C and 7D). These results suggest that at the functional level, PF-05089771 does not impact iPSC nociceptor excitability in the absence of Na_V_1.7.

## Discussion

We have found that CIP in humans is due to a profound peripheral phenotype characterized by hypoexcitability of nociceptive afferents but also important structural changes in these afferents. We have used human iPSC-derived nociceptors to study CIP at the cellular level and have shown that Na_V_1.7 is localized to specialized neuronal compartments within nociceptors and is critical in regulating excitability. We demonstrated that this cellular model is helpful for probing selectivity of clinical compounds aimed at inhibiting Na_V_1.7 and alleviating pain.

All CIP participants we studied are compound heterozygotes for *SCN9A* mutations that drastically reduced the Na_V_1.7 current. The R896W, R830X, and G1725R mutations were associated with almost total loss of channel function, while the FS1773 mutation, which resides in the C terminus of the channel, resulted in an 8-fold reduction in the current density. The majority of previously characterized CIP Na_V_1.7 mutations result in complete loss of channel function (Bennett and Woods, 2014); however, one other recent paper has also reported a number of CIP-associated *SCN9A* mutations in which some current is retained (albeit significantly reduced) (Emery et al., 2015). The FS1773 allele was paired (as a compound heterozygote) with R830X, in which no current was observed. Therefore, very little current is likely to be generated *in vivo*.

All participants had a clinical presentation consistent with CIP secondary to bi-allelic *SCN9A* mutations. They had not experienced pain at any point in their lives or during our QST and algogen testing and were anosmic. The sensory deficits were not restricted to pain perception; participants were also hyposensitive to warming and, to a lesser extent, cooling and had impaired thermal sensory limen. These modalities are mediated by C and Aδ thermoreceptors. It has previously been reported that the flare response to histamine is preserved in these patients (Goldberg et al., 2007), a finding that we replicated here. Previously unappreciated, however, is that although the neurogenic flare is preserved, this stimulus does not evoke a perception of itch in participants. This likely reflects the fact that there is a large overlap in the primary afferent populations responding to algogens and pruritogens, the majority of which express Na_V_1.7 (Schmelz et al., 2003, Usoskin et al., 2015). In preclinical models of itch behavior, animals treated with a function-blocking antibody to Na_V_1.7 demonstrated less of a response to pruritic stimuli (Lee et al., 2014), and a recent report detailing adult-onset ablation of Na_V_1.7 demonstrated a loss of itch behaviors in response to histamine and chloroquine (Shields et al., 2018). Large fiber-mediated modalities, such as detection of vibration and light touch, were normal.

We undertook a number of electrophysiological tests studying the impact of Na_V_1.7 mutations on sensory nerve function *in vivo*. VGSC mutations, as well as VGSC binding drugs and toxins, have been shown to alter nerve excitability parameters in humans (Kiernan et al., 2005a, Kiernan et al., 2005b). We therefore undertook sensory nerve excitability testing but did not find any significant changes in the patients. The likely reason for this is that sensory nerve action potentials (used as the outcome measure for threshold tracking) are dominated by activity of large myelinated axons. In rodents, only 15% of medium and large diameter myelinated DRG neurons were reported to be Na_V_1.7 immunoreactive (Black et al., 2012). Furthermore, these neurons express other TTX-s VGSCs such as Na_V_1.1 and Na_V_1.6. This finding suggests that nerve excitability parameters are unlikely to be a helpful biomarker in clinical trials of Na_V_1.7 blockers. We therefore proceeded to microneurography in which activity of small-diameter sensory and sympathetic axons, C- and Aδ-fibers, can be directly assessed.

The findings on microneurography of the superficial peroneal nerve were striking. Small fibers with the characteristics of C- and Aδ-low-threshold thermoreceptors, as well sympathetic efferents, could be readily recorded and identified based on the profile of ADS of conduction velocity and natural activation using previously well-established criteria (Serra et al., 1999, Serra et al., 2004, Campero et al., 2004). However, we did not record any units with the characteristics of C-fiber nociceptors. In healthy volunteers, the proportion of identified C-nociceptors using the same searching technique as the one employed in this study amounts to roughly 60% of all C-fibers in the superficial peroneal nerve (Serra et al., 1999, Campero et al., 2004). It is important to note that electrical stimulation applied to the innervation territory of the superficial peroneal nerve was used as a search strategy, therefore ruling out a hypothetical “search bias” if natural stimuli would have been used. One possibility for the lack of C-nociceptor “profiles” would be that C-nociceptor axons were, in fact, present but rendered unexcitable by the mutation; therefore, we would have not been able to detect them with microneurography. This hypothesis would be consistent with our findings in iPSC nociceptors of hypoexcitability. An alternative explanation would be that there is an anatomical lack of C-nociceptors, which is a hypothesis that we explored with skin biopsy.

The dogma has been that small fibers are structurally normal in CIP due to *SCN9A* mutations. The nerve biopsy in the original *SCN9A* pedigree was normal (Cox et al., 2006). This is thought to distinguish CIP due to *SCN9A* mutations from CIP due to other causes, for example, NGF mutations (Einarsdottir et al., 2004), which result in severe developmental degeneration of nociceptors. To our surprise, there were clear and marked reductions in epidermal innervation of the distal leg in all three of the *SCN9A* CIP participants. IENFD in these participants was well below the fifth centile of normative data for age and gender. Two previous case reports have also reported reduced IENFD when assessed at a distal site (Nilsen et al., 2009, Marchi et al., 2018).

One explanation for the reduction of IENFD is the outcome of previous injuries. We therefore also performed skin biopsies from the thigh, a more proximal site with a higher innervation density than the distal leg. Again, there was a virtual absence of epidermal nerve fibers in the CIP patients. Dermal nerve fibers could still be observed. The implication is that in humans, Na_V_1.7 is required for the long-term structural integrity of the distal terminations of nociceptors. A recent case report describes a young patient (2-year-old girl) with CIP due to *SCN9A* mutations, in which IENFD was already severely reduced (Marchi et al., 2018). The loss of IENFs in CIP could be due to a “dying-back” in early life stages or disruption of normal C-nociceptor development during embryogenesis, occurring as a result of the loss of Na_V_1.7 function. Our studies *in vitro* failed to observe an intrinsic defect in neurite outgrowth of iPSC nociceptors lacking Na_V_1.7. However, appropriate epidermal targeting of endogenous nociceptive afferents requires complex environmental and chemical cues and for axons to travel large distances (Wang et al., 2013), which we cannot fully model in our culture system. Therefore, we cannot rule out a role for loss of Na_V_1.7 in this context. The contribution of Na_V_1.7 to IENFD could be due to afferent hypoexcitability (discussed below) or a non-canonical role for the ion channel.

Our human data contrast with findings in mice lacking Na_V_1.7, in which epidermal innervation is normal (Gingras et al., 2014). The reason for the loss of IENFs in humans, but not mice, is not resolved; there could be differences in activity-dependent maintenance of C-fiber terminals between the two species, especially given the need to maintain longer axons in human.

The presence of a normal flare reaction despite complete analgesia and virtually complete denervation of the epidermis is intriguing. The flare response is neurally mediated following activation of cutaneous C-fibers, leading to axon reflex-mediated release of neuropeptides onto the perforating arterioles of the skin (Szolcsanyi et al., 1992, Meyer et al., 1985). If all C-fibers had degenerated, then the flare response would be absent. Its preservation suggests that at least some C-fiber terminals lacking Na_V_1.7 within the dermis could still maintain short-range action propagation (sufficient to induce neuropeptide release), even if they were unable to continue such conduction to the nerve trunk.

These *in vivo* findings predict that following application of noxious stimuli to CIP patients, there will be a marked reduction of nociceptor input from the periphery to the spinal cord and subsequently to higher brain centers. In one participant, we undertook fMRI using arterial spin labeling to test this hypothesis. Using a previously published protocol (Segerdahl et al., 2015), we applied the algogen, capsaicin, and warmth to the leg. This did not elicit any pain in the CIP participant but was intensely painful in control participants. Increased CBF was observed in the primary somatosensory cortex, the dorsal anterior cingulate, and the posterior cingulate in the CIP participant. However, in healthy controls, a much more extensive cortical network demonstrated significant increases in CBF that included primary and secondary somatosensory cortices, dorsal lateral prefrontal cortex, insula (anterior, mid, and posterior), anterior cingulate, putamen, nucleus accumbens, periaqueductal gray, and the cerebellum. The NPS is an fMRI-based measure that has been shown to be predictive of pain intensity (Wager et al., 2013). A comparison of the NPS response values showed that there was no overlap between the NPS and brain activity from the CIP participant in response to capsaicin-induced tonic heat hyperalgesia. By comparison, the strong NPS response in HCs reflects that there was clear overlap with the NPS and pain-related brain activity in these participants, and this was strongly related to an intense pain experience; data that were confirmed by the pain reports collected during these scans. One previous study did not find a difference in BOLD fMRI responses within *a priori*-defined regions of interest identified using Neurosynth (that does not restrict regions to nociceptor-driven pain) when comparing CIP patients with healthy controls (Salomons et al., 2016). However, their approach has been criticized from a methodological and interpretative perspective, limiting the conclusions to be drawn (Büchel et al., 2016). There are a number of key differences between the Solomons study and ours that limit direct comparison, including the imaging method, pain modality, and analysis pipeline employed. Crucially, we used a tonic application of the algogen capsaicin, which is well known to evoke moderate-to-severe pain in healthy controls by selectively activating nociceptors and is not confounded by co-activation of low-threshold mechanoreceptors. Nonetheless, we conclude here that a lack of nociceptor-driven pain in CIP does not result in the same activation pattern as healthy controls and, as such, supports the peripheral observations.

In order to better understand the impact of Na_V_1.7 mutations on the excitability of sensory neurons, we used iPSC-derived nociceptors combined with genome engineering. We first characterized the expression and sub-cellular localization of Na_V_1.7 in iPSC nociceptors. Na_V_1.7 was robustly expressed at the soma membrane, along the length of the axon, and was enriched in terminal structures. In myelinated axons, the channel was appropriately localized to nodes of Ranvier as previously reported *in vivo* in rodent (Black et al., 2012).

Consistent with studies of rodent Na_V_1.7 KO DRGs (Raouf et al., 2012), we observed reliable hypoexcitability in CIP iPSC nociceptors, demonstrable by an increased current threshold to activation and a reduction in action potential firing in response to prolonged supra-threshold stimulation. These phenotypes replicated in isogenic Na_V_1.7 KO iPSC nociceptors and rescued in CIP iPSC nociceptors in which one deleterious *SCN9A* allele was genome corrected to the wild-type sequence. Interestingly, while firing thresholds were completely reversed to normal in our genome-corrected iPSC nociceptors, firing in response to supra-threshold stimuli was not. This may indicate that human nociceptors require two functional copies of *SCN9A* to be fully excitable.

The isogenic Na_V_1.7 KO iPSC nociceptors allowed us to further interrogate the association between channel activity and nociceptor function. In contrast to a recent report suggesting a limited contribution of Na_V_1.7 to total Na_V_ current in human sensory neurons (Zhang et al., 2017), we found that the channel provides ∼24% of peak total Na_V_ current in our iPSC nociceptors.

Zhang et al. (2017) failed to record TTX-sensitive low-threshold ramp currents in cadaveric human DRG *in vitro* and had mixed results when using selective toxins and blockers to assess the contribution of Na_V_1.7 to total TTX-sensitive currents. Pro-Tx II failed to inhibit putative TTX-s currents, while PF-05089771 resulted in complete inhibition. The authors postulate that either Pro-Tx II loses potency or PF-05089771 loses selectivity when used against Na_V_ channels in their native environment rather than heterologous expression systems where both have previously been shown to be potent and specific (Schmalhofer et al., 2008, Alexandrou et al., 2016). Our data would argue that, at least in terms of functional outcome (i.e., firing properties), PF-05089771 is selective for Na_V_1.7. This conclusion complements our findings of consistent hypoexcitability in iPSC lines lacking Na_V_1.7 function and leads us to conclude that the channel contributes significantly to neuronal properties. At the level of transcription, Na_V_1.7 is the most abundantly expressed Na_V_ channel in human DRG (Chang et al., 2018), and it would be surprising if the channel did not play a significant role in sensory neuron physiology. Dissociation and culture of cadaveric DRG and our iPSC differentiation strategy offer two approaches to study human sensory neuron biology. iPSC differentiation results in neurons that share many transcriptional and functional characteristics of *in vivo* nociceptors (Chambers et al., 2012, Young et al., 2014), but it does not recapitulate the whole panoply of sensory neurons present in the DRG. Cadaveric DRG are becoming more available, but the study of very rare diseases, such as CIP, will remain a challenge. Genetic modification of iPSCs allows the refined interrogation of gene function, negating the confound of compound selectivity inherent to pharmacological studies.

We have consistently found that iPSC nociceptors that lack Na_V_1.7 (whether derived from CIP or Na_V_1.7 KO lines) were still able to generate action potentials at the cell soma (albeit requiring greater current stimuli). This finding is consistent with computer simulation models and dynamic clamp recordings, which describe a linear relationship between Na_V_1.7 conductance and the threshold for firing a single action potential, as well as firing in response to supra-threshold depolarizing stimuli (Choi and Waxman, 2011, Vasylyev et al., 2014). Crucially in these studies, as in ours, nociceptors are capable of generating action potentials at the cell soma in the complete absence of Na_V_1.7 conductance.

Channel kinetics of Na_V_1.7 position it as an attractive candidate to amplify small depolarizing currents and to mediate action potential electrogenesis at the peripheral terminal (Cummins et al., 1998). We attempted to better model terminal generator potentials *in vitro* by applying a graded supra-threshold stimuli that slowly depolarized the membrane potential (Cheng et al., 2011). Na_V_1.7 KO iPSC nociceptors were markedly less able to respond than healthy control iPSC nociceptors, especially in response to slower depolarizing stimuli. A necessary role for Na_V_1.7 in initial generation and invasion of the action potential in nociceptor terminals would be consistent with our *in vivo* microneurography findings. There are preclinical data that Na_V_1.7 may regulate neurotransmitter release from the central terminal of nociceptors (Alexandrou et al., 2016, Minett et al., 2012). This is certainly a plausible mechanism, although it is unlikely to make a major contribution to lack of pain perception in the participants with CIP that we report here, given the lack of functional nociceptor axons within peripheral nerve, such that the deficit in nociceptive transmission is proximal to the central terminal.

We took advantage of our Na_V_1.7 KO to probe pharmacology. Although endogenous opioids have been suggested to be upregulated in the absence of functional Na_V_1.7 and to contribute to the CIP phenotype (Minett et al., 2015), we did not see upregulation of PENK mRNA in Na_V_1.7 KO iPSC nociceptors or meaningful expression to begin with in healthy control neurons. In support of this finding, the marked hypoexcitability in Na_V_1.7 KO iPSC nociceptors that we observed was independent of endogenous opioid signaling. These results in isolated iPSC nociceptors, however, cannot rule out a role for central opioid mechanisms contributing to analgesia in the absence of Nav1.7. We utilized our Na_V_1.7 KO lines to test the specificity of Na_V_1.7 selective small molecules in clinical development. PF-05089771 is a Na_V_1.7-selective arylsulfonamide that has undergone phase II trials for primary erthromyelgia (Cao et al., 2016) and painful diabetic neuropathy (McDonnell et al., 2018). Treatment of healthy control iPSC nociceptors with PF-05089771 decreased excitability, whereas it had no effect on Na_V_1.7 KO neurons, suggestive of good specificity. BII074 has successfully undergone phase II trials for trigeminal neuralgia (Zakrzewska et al., 2017). We found Na_V_1.7-independent effects present at clinically relevant concentrations that enhanced steeply as dose increased. In particular, use-dependent block of nociceptor firing by BII074 appeared to be largely independent of Na_V_1.7. This may be particularly relevant for trigeminal neuralgia, in which there are paroxysms of pain. This drug was well tolerated in the phase II trial (Zakrzewska et al., 2017); however, our results would caution against seeking to increase dosage to enhance therapeutic outcomes due to the risk of off-target effects.

To summarize, we have undertaken a detailed assessment of the clinical phenotype of CIP due to *SCN9A* mutations as well as investigating the impact of these mutations on both cellular models and the somatosensory nervous system *in vivo*. The sensory impairments due to loss of Na_V_1.7 do not only relate to pain, but subjects also demonstrate deficits in temperature discrimination and itch. Na_V_1.7 is expressed by human iPSC nociceptors, is trafficked to the cell surface, axon, and terminals, and has a key role in the regulation of excitability especially in response to slow-graded depolarizing stimuli. Reassuringly, recently developed Na_V_1.7 blockers tested in these cellular models were able to alter somal excitability to levels similar to that of Na_V_1.7 KO, with a caveat that in some cases these drugs lacked specificity for Na_V_1.7. Finally, in humans with bi-allelic *SCN9A* mutations, we have found a profound loss of nociceptors *in vivo* defined both structurally and functionally. This likely reflects the long-term loss of Na_V_1.7 and may suggest that acute pharmacological inhibition of Na_V_1.7 in humans may not fully replicate the CIP phenotype.

## STAR★Methods

### Key Resources Table

**Table:** 

REAGENT or RESOURCE	SOURCE	IDENTIFIER
**Antibodies**		

Rabbit anti-Brn3a	Millipore	Cat# AB5945; RRID: AB_92154
Chicken anti-NF200	Abcam	Cat# ab4680; RRID: AB_304560
Rabbit anti-HA	Cell Signaling Technology	Cat# 3724; RRID: AB_1549585
Mouse anti-HA	Sigma-Aldrich	Cat# A2095; RRID: AB_257974
Mouse anti-Beta-Actin	Sigma-Aldrich	Cat# A5316; RRID: AB_476743
Rat anti-MBP	Abcam	Cat# ab7349; RRID: AB_305869
Phalloidin-Tetramethylrhodamine B isothiocyanate (TRITC)	Sigma-Aldrich	Cat: P1951; RRID: AB_231514
Rabbit anti-Protein Gene Protein 9.5 (PGP 9.5)	Zytomed	Cat# 516-3340
Amersham ECL Mouse IgG, HRP-linked whole antibody (from sheep)	GE Healthcare Life Sciences	Cat# NA931V; RRID: AB_772210
Amersham ECL Rabbit IgG, HRP-linked whole antibody (from donkey)	GE Healthcare Life Sciences	Cat# NA934V; RRID: AB_772206
Donkey anti-rabbit IgG Alexa 488	Thermo Fisher Scientific	Cat# A-21206; RRID: AB_2535792
Donkey anti-rabbit IgG Cy3	Jackson ImmunoResearch Labs	Cat# 711-166-152; RRID: AB_2313568
Donkey anti-mouse IgG Alexa 488	Thermo Fisher Scientific	Cat# A-21202; RRID: AB_141607
Goat anti-chicken Alexa 488	Thermo Fisher Scientific	Cat# A-11039; RRID: AB_2534096
Donkey anti-rabbit IgG Alexa 546	Thermo Fisher Scientific	Cat# A10040; RRID: AB_2534016
Goat anti-rabbit IgG Alexa 488	Thermo Fisher Scientific	Cat# A-11008; RRID: AB_143165
Guinea pig anti-CASPR	Gift from Bhat MA	N/A
Mouse anti-S100	Sigma-Aldrich	Cat#S2532; RRID: AB_477499
Goat anti-Rat CF405M	Sigma-Aldrich	Cat#SAB4600463
Biotinylated Goat anti-rabbit	Vector Biolabs	Cat# BA-1000; RRID: AB_2313606
Alexa Fluor 488 streptavidin	Thermo Fisher Scientific	Cat#S11223; RRID: AB_2336881

**Bacterial and Virus Strains**		

Lentivirus CAMKII-EGFP	Signagen	Cat#: SL100304

**Chemicals, Peptides, and Recombinant Proteins**		

BDNF recombinant human	Thermo Fisher Scientific	Cat#10908010
NT3 recombinant human	Peprotech	Cat#450-03
β-NGF recombinant human	Peprotech	Cat#450-01
GDNF recombinant human	Peprotech	Cat#450-10
Cultrex Mouse Laminin I, Pathclear	R&D Systems	Cat#3401-010-02
CHIR99021	Sigma-Aldrich	Cat#SML1046
SU-5402	Sigma-Aldrich	Cat#SML044
DAPT	Sigma-Aldrich	Cat#D5942
LDN-193189	Sigma-Aldrich	Cat#SML0559
SB431542	Sigma-Aldrich	Cat#616461
PF-05089771	Sigma-Aldrich	Cat#PZ0311
BIIB074	Axon Medchem	Cat#2548
Naloxone	Sigma-Aldrich	Cat#N7758

**Critical Commercial Assays**		

Pierce BCA Protein Assay Kit	Thermo Fisher Scientific	Cat#: 23227

**Deposited Data**		

Human iPSC (AD2, AD3, NHDF, AH017) and iPSC-nociceptor RNA-seq data	Baskozos et al., 2019	GEO: GSE107181
Human DRG L2 RNA-seq data	Ray et al., 2018	dbGAP: phs001158.v1.p1
Single cell RNA-seq human iPSC and iPSC-nociceptor data	Schwartzentruber et al., 2018	ENA: ERP020576
Human whole blood, skin, skeletal muscle, fibroblasts, and tibial nerve raw counts	GTEx V7	N/A

**Experimental Models: Cell Lines**		

Human iPSC line_ HC1	StemBANCC Consortium	AD2-1
Human iPSC line_ HC2	StemBANCC Consortium	NHDF-1
Human iPSC line_HC3	StemBANCC Consortium	AH017-7
Human iPSC line_CIP1.1	StemBANCC Consortium	811-05-01
Human iPSC line_CIP1.2	StemBANCC Consortium	811-05-03
Human iPSC line_CIP2	StemBANCC Consortium	246-03-01

**Oligonucleotides**		

YWHAZ F 5′-CCTGCATGAAGTCTGTAACTGAG-3′	IDT	N/A
YWHAZ R 5′-GACCTACGGGCTCCTACAACA-3′	IDT	N/A
SCN9A F 5′-GGCATAGGCGAGCACATGAA-3′	IDT	N/A
SCN9A R 5′-AACAAGGAGCCACGAATGCT-3′	IDT	N/A

**Recombinant DNA**		

pSpCas9(BB)-2A-Puro (PX459) V2.0	Addgene (Depositor:Feng Zhang)	#62988

**Software and Algorithms**		

ImageJ/Fiji	NIH	https://imagej.nih.gov/ij/index.html, https://fiji.sc/
Clampfit 10	Molecular Devices	http://mdc.custhelp.com/app/answers/detail/a_id/18779/?/axon%E2%84%A2-pclamp%E2%84%A2-10-electrophysiology-data-acquisition-%26-analysis-software
Prism 7.0	GraphPad software	https://www.graphpad.com/
FMRIB’s Software Library	Smith et al., 2004	https://fsl.fmrib.ox.ac.uk/fsl/fslwiki
QTRAC software	Institute of Neurology, London, UK	N/A
STAR version 2.5.2b	Dobin et al., 2013	https://github.com/alexdobin/STAR
HTSeq version 0.11.0	Anders et al., 2015	https://github.com/simon-anders/htseq
DESeq2 version 1.18.1	Love et al., 2014	https://www.bioconductor.org/packages/release/bioc/html/DESeq2.html
GTEx portal version 7	Carithers and Moore, 2015	https://gtexportal.org/home/
R version 3.4.4	R Development Core Team, 2018	https://cran.r-project.org/
Ggplot2 version 2.2.1	Wickham, 2009	https://cran.r-project.org/web/packages/ggplot2/index.html
IN Cell Developer Toolbox analysis software build 1.9.2	GE	IN Cell Developer Toolbox analysis software build 1.9.2

### Contact for Reagent and Resource Sharing

Further information and requests for resources and reagents should be directed to and will be fulfilled by the Lead Contact, Professor David Bennett (david.bennett@ndcn.ox.ac.uk).

### Experimental Model and Subject Details

#### Ethics

The three study participants (31 year old male, 34 year old male and 44 year old female) signed written consent as part of the Painful Channelopathies Study, approved by Riverside research ethics committee (NRES reference: 12/LO/0017).

#### Generation and Culture of hiPSC Lines

Healthy control iPSCs were derived from fibroblasts as described in Clark et al. (2017). AD2 (termed HC1 throughout the study) from 51 year old male, was reprogrammed by non-integrating Sendai viral vectors using the CytoTune-iPS Reprogramming Kit (ThermoFisher). NHDF (termed HC2 throughout the study) from 44-year-old female, was reprogrammed with retroviral vectors (Addgene: 17220: pMXs-hc-MYC, 17219: pMXs-hKLF4, 17218: pMXs-hSOX2, 17217: pMXs-hOCT3/4, 13354: pMXs-Nanog). AH017-7 (termed HC3 throughout the study) from 67-year-old female, was reprogrammed using the tetracistronic Sendai virus vector SeVdp(KOSM)302L. CIP iPSC patient lines were obtained through the IMI/EU sponsored StemBANCC consortium via the Human Biomaterials Resource Centre, University of Birmingham, UK (https://www.birmingham.ac.uk/facilities/hbrc). Fibroblasts from CIP 1 (31-year-old male) and CIP 2 (34-year-old male) were reprogrammed using the CytoTune-iPS Reprogramming Kit (ThermoFisher) to generate two clones from CIP 1 (cCIP1.1, and cCIP1.2) and one from CIP 2 (cCIP2). All iPSC lines were subject to strict quality control checks before initiation of differentiation and following genome editing. This included CytoSNP analysis (Illumina CytoSNP-12-v2.0 array) and pluripotency characterization. All cells were karyotypically normal and negative for mycoplasma. iPSC were maintained in mTesR1 (StemCell Technologies) on Matrigel (Corning) coated dishes. Cells were routinely passaged at 80% confluence with either Versene EDTA or Accutase treatment (Life Technologies). In the event single cells were required re-plating, medium was supplemented with Y-27632 (Tocris).

#### Differentiation of iPSCs

iPSCs were differentiated following a previously published protocol (Chambers et al., 2012) with modifications. In brief, cells were passaged using Versene EDTA (ThermoFisher) and plated at high density. Neural induction commenced with the addition of SMAD inhibitors SB431542 (Sigma, 10 μM) and LDN-193189 (Sigma, 100 nM) to KSR base medium (Knockout-DMEM, 15% knockout-serum replacement, 1% Glutamax, 1% nonessential amino acids, 100 μM β-mercaptoethanol, (ThermoFisher)). Three additional small molecules were introduced on day 3 (CHIR99021 (Sigma, 3 μM), SU5402 (R&D Systems, 10 μM) and DAPT (Sigma, 10 μM). The dual SMAD inhibitors were withdrawn on day 5. The base medium was gradually transitioned to N2/B27 medium (Neurobasal medium, 2% B27 supplement, 1% N2 supplement, 1% Glutamax, (ThermoFisher)) in 25% increments. Cells were replated onto glass coverslips at day 12 of the differentiation in N2/B27 medium supplemented with four recombinant growth factors at 25ng/ml (BDNF; ThermoFisher, NT3, NGF, GDNF; Peprotech). CHIR90221 was included for 4 further days. Laminin (Cultrex Mouse Laminin I, R&D systems, 500ng/ml) was included in long-term maintenance medium from 25 days onward. Medium changes were performed twice weekly.

### Method Details

#### Neurological assessment

Each study participant underwent a comprehensive structured neurological examination. A detailed upper and lower limb neurological examination was performed to detect clinical signs of a peripheral neuropathy (O’Brien, 2010). Orthostatic hypotension was assessed by measuring lying and standing blood pressure and was defined as either a 20 mm Hg reduction in systolic or a 10 mm Hg reduction in diastolic blood pressure within 3 min of standing.

#### Nerve conduction tests

Nerve conduction tests were performed with an ADVANCE system (Neurometrix, Massachusetts, USA) and used conventional reusable electrodes. Sensory nerve conduction studies were recorded from the sural, superficial peroneal, median and ulnar nerves. Motor nerve conduction studies were recorded from the peroneal, tibial, median, and ulnar nerves. The minimum case definition criterion for electrodiagnostic confirmation of peripheral neuropathy was an abnormality of any attribute of nerve conduction in two separate nerves, one of which was the sural nerve (Buschbacher and Orahlow, 2006). Variables such as skin temperature, age, height, sex, and weight were measured and accounted for when interpreting nerve conduction tests. Our protocol was in line with those recommended by the American Academy of Neurology and American Association of Electrodiagnostic Medicine. Nerve conduction tests were not repeated if study participant had previous results.

#### Nerve excitability testing

Sensory nerve excitability measurements were performed using automated QTRAC software (TRONDNF, QTRAC, Institute of Neurology, London, UK). This protocol was designed for the rapid acquisition of multiple excitability parameters (Kiernan et al., 2000). Sensory nerve action potentials were recorded from the index finger using surface electrodes after stimulation of the median nerve at the wrist. Skin temperature was monitored near the site of stimulation and maintained above 32°C for each study. Each nerve excitability recording consists of four tests that include stimulus–response behavior, threshold electrotonus, the recovery cycle and the current–threshold relationship (Kiernan et al., 2000). Nerve excitability measurements provide indirect information about the behaviors of voltage-gated sodium channels, potassium channels, energy-dependent pumps and exchangers that are activated during the process of action potential generation and impulse transmission (Krishnan et al., 2009).

#### Intra-epidermal nerve fiber assessment

The determination of intra-epidermal nerve fiber density (IENFD) from skin biopsy samples is a validated and sensitive diagnostic tool for the assessment of small fiber pathology. Biopsy samples were taken in accordance with the consensus document produced by the European Federation of Neurological Societies/Peripheral Nerve Society Guideline on the utilization of skin biopsy samples in the diagnosis of peripheral neuropathies (Lauria et al., 2010). Skin biopsies were taken with a disposable 3mm punch biopsy circular blade (Stiefel Laboratories Inc, GSK Plc) from 10 cm proximal to the lateral malleolus and from the lateral aspect of the proximal thigh. Nerve fibers were visualized using rabbit anti-PGP9.5 antibody (Zytomed, 1:200) with Cy3-conjugated donkey anti-rabbit IgG (Jackson ImmunoResearch, 711-165-152, 1:1000). The nerve fibers are counted as they cross the epidermal-dermal junction in order to quantify intra-epidermal nerve fiber density. Images were taken using an LSM 700 microscope with a Plan-Apochromat objective (Carl Zeiss) at 40 × and 63 × magnification.

#### Quantitative sensory testing (QST)

Somatosensory phenotype was determined using a published protocol of the German research network of neuropathic pain (DFNS) (Rolke et al., 2006). Cold and warm detection thresholds, as well as cold and heat pain thresholds and thermal sensory limen (including paradoxical heat sensations), were established using a Thermotest (Somedic, Hörby, Sweden). We also tested mechanical detection and pain thresholds as well as mechanical pain sensitivity, allodynia, pressure pain thresholds, wind up ratio and vibration detection thresholds. Participants were familiarized with the testing procedure on the dorsum of the forearm before all parameters were measured over the dorsum of hand and foot. Pressure pain thresholds were recorded over the thenar eminence and arch of the foot. Vibration detection thresholds were tested over the ulnar styloid and medial malleolus.

QST data were entered into the data analysis system Equista provided by the DFNS. Equsita transformed the raw QST data into z-scores thus normalizing for age, gender, and body location of testing (Magerl et al., 2010). Positive z-scores denote gain of function whereas negative z-scores denote loss of function. We had previously generated z-scores for QST measures included in the DFNS protocol for participant CIP1 (Ramirez et al., 2014). These have been replotted using the latest version of Equista in order to generate z-scores which are comparable between the three participants.

#### Chemical algogens

We applied 30% mustard oil (Allyl isothiocyanate dissolved in olive oil, Sigma) to the volar surface of the forearm at a midpoint between the wrist and elbow. We iontophoresed 2% histamine (0.02 g in 100ml 0.9% NaCl, Sigma) into the skin of the volar surface of the forearm at a midpoint between the wrist and elbow, at 1.2mA for 20 s.

#### Microneurographic recordings

Microneurography (Vallbo and Hagbarth, 1968) was used to record action potentials of C-fibers from the lateral branch of the superficial peroneal nerve at the dorsum of the left foot. The subjects sat relaxed on a recliner, with the legs supported on a padded platform. Intraneural recordings were obtained using a 1MΩ impedance, 200μm-diameter lacquer-insulated tungsten microelectrode (FHC, USA), which was inserted percutaneously into the nerve. An uninsulated tungsten reference electrode was inserted into the skin 1 to 2 cm outside the nerve trunk. Neural signals were amplified by an isolated high-input impedance differential amplifier (NeuroAmpEx; ADInstruments, Australia) and filtered with an adjustable analog filter (gain 10,000; band-pass 100-2,000Hz). To improve the recorded signal quality, 50Hz mains line interference was removed with an on-line noise eliminator (Hum Bug, Quest Scientific, Canada). The recorded and amplified nerve signal was digitized (NI DAQCARD-6062E; National Instruments Europe Corp., Debrecen, Hungary) at a sampling rate of 20 kHz. Further digital filtering (band-pass 300–2,000 Hz) and clamping of the baseline were performed both on-line and during offline analysis for a better visualization of the action potentials. Skin temperature was recorded continuously with an infrared thermometer (PCE-IR10, PCE Iberica, Spain) pointing to the skin adjacent to the receptive fields of the units under study. Responses were recorded and analyzed with QTRAC software (Institute of Neurology, London, UK), specially modified to track peak latencies and display them as a raster plot. In the latency raster plots, each peak in the filtered voltage signal that exceeded a specified level is represented by a dot on a plot with latency as the ordinate and elapsed time as the abscissa (see Serra et al., 1999). Depending on the level chosen, the dots could represent action potentials or noise. An isolated constant-current stimulator (DS7; Digitimer Ltd, UK) was used for stimulation (rectangular pulses, 300μs duration) of the cutaneous receptive fields with a pair of needle stainless steel electrodes resting on the surface of the skin. Only fibers with latencies compatible with conduction velocities in the C-fiber range (< 2 m/s) were studied. A combination of 0.25-Hz baseline stimulation and a 3-min 2-Hz train were given to induce activity-dependant slowing (ADS) of conduction velocity. Profiles of ADS were used to classify the recorded C-fibers into C-nociceptors, C-thermoreceptors, low threshold C-mechanoreceptors or sympathetic efferents following pre-established criteria (Serra et al., 1999, Serra et al., 2004, Campero et al., 2001, Campero et al., 2004).

#### fMRI

The CIP participant (CIP 2) was scanned on two visits (1 week apart) using a 3T Siemens scanner fitted with a 32-channel head and body coil. Each scan visit was 2 h. T1-weighted structural images were acquired with a 3D MPRAGE sequence (1 × 1 x 1 mm voxels). Absolute cerebral blood flow (CBF) data were acquired using a multi-inversion time pseudo-continuous arterial spin labeling (pCASL) sequence described previously (Segerdahl et al., 2015). Briefly, ‘tag’ and ‘control’ images were acquired sequentially every repetition time (TR = 4 s) with a label duration of 1.4 s. A total of six inversion times were used. B0 shimming was employed to mitigate off-resonance artifacts within the imaging region and labeling plane. A total of 96 volumes were analyzed for each experimental condition.

The participant was scanned during four different conditions: rest (no stimulation), tonic thermal stimulation (T = 40.3C), tonic capsaicin, and the tonic thermal stimulation applied to the capsaicin-treated skin (“thermal + capsaicin”). Stimulations were applied to the antero-medial aspect of the lower right forearm. The capsaicin scans did not commence until the participant’s skin was treated with the cream for 25 min. This period was found previously to capture of the onset of 1% capsaicin-induced heat pain (Segerdahl et al., 2015). Three 5-min runs of each condition were scanned in each session (Figure 4B). Verbal intensity ratings (using an 11-point numerical rating scale) were collected at the start and end of each scan run. For comparison, data collected from twelve healthy control participants (HC) experiencing a similar topical capsaicin cream paradigm (Segerdahl et al., 2015) were included in our analysis pipeline for comparison with the CIP participant data. Here, each healthy control participant was scanned once. During this scan, a single trial testing the effects of each experimental condition once was observed for that subject. Multiple-repeat trials were not completed on the HC cohort.

All data were analyzed using FMRIB’s Software Library (FSL) (Smith et al., 2004). The FMRI analysis was completed in each subject’s native anatomical space and then was co-registered to a standard MNI152 template brain using non-linear registration (FNIRT). ASL functional data were pre-processed using previously published methods that adhere to current guidelines (Alsop et al., 2015, Okell et al., 2013, Segerdahl et al., 2015). The absolute CBF time series generated for each subject during each condition was averaged using a mixed effects model (to account for voxel-wise variance of the Bayesian fit during CBF quantification). This generated a whole brain voxel-wise absolute CBF volume with a corresponding variance image for each scan run at each condition to use in FEAT. A total of six runs (i.e., 3 scan runs X 2 scan sessions) were collected from the CIP patient for each condition. The whole brain absolute CBF volumes were then inputted into a repeated-measures design in FEAT to determine the average change in perfusion for each condition compared to rest (Mixed Effects; z > 3.1, p < 0.05) (Figure 4C).

Additionally, we used the Neurological Pain Signature (NPS) to test whether the perfusion changes observed during the capsaicin-evoked thermal condition were overlapping anatomically with this physical pain signature thereby confirming this was a distinct perceptual experience in CIP versus HC. The “thermal+capsaicin > rest” condition was selected because it was the maximally salient condition experienced by both groups (albeit a non-painful one for CIP) (Figure 4A) and it was the only contrast to yield a statistically significant change in perfusion in CIP (Figure 4C). To do this we applied the NPS to the subject-level cope images representing the contrast between the “thermal + capsaicin” and “rest” conditions using the CanLab Toolbox (Wager et al., 2013). Briefly, the NPS is a multivariate pattern of brain activity that is both sensitive and specific for classifying phasic and acute experimental physical pain from non-painful stimuli and can be used as a proxy for measuring heat pain intensity (Wager et al., 2013). The NPS was calculated for each cope image by taking the dot product of it and the NPS. The mean scalar NPS values for both CIP (i.e., mean across six repeat trials) and HC (mean across the group) are plotted in (Figure 4D).

#### Plasmids and site-directed mutagenesis

Human Na_V_1.7 cDNA was cloned into a modified pcDNA3 expression vector containing downstream IRES and dsRED2 sequences (SCN9A-IRES-DsRED) (Cox et al., 2006). Human β1 and β2 subunits were cloned into pIRES2-AcGFP (SCN1B-IRES-SCN2B-IRES-eGFP) (Cox et al., 2006). Mutations were introduced using QuikChange II XL site-directed mutagenesis kit (Agilent).

#### CRISPR-Cas9 genome editing

*Streptococcus pyogenes* Cas9 target sites were identified using the online CRISPR design tool (https://crispr.mit.edu) or ChopChop (http://chopchop.cbu.uib.no/). Two to three guides were selected per location and *in vitro* cutting was assessed using HEK293T cells. Finalised gRNAs were selected based on strength of T7E1 assay (New England Biolabs) and proximity to the desired editing loci. iPSCs were dissociated with Accutase, resuspended in mTesR1 supplemented with Y-27632 (Tocris). Dissociated cells were then immediately transfected with 6 μg of PX459 pSpCas9(BB)-2A-Puro V2.0 (Addgene) and 2 μg of phosphorothioate-treated ssODN (Integrated DNA Technologies) using LT-1 (Mirusbio) reagent. Puromycin (0.3 μg/ml to 0.35 μg/ml) (ThermoFisher) was added to the cells 18 h post-transfection for 48-72 h. Following selection, cells were plated at limiting densities for single clone isolation. Isolated iPSC colonies were manually dissected and picked using a 21G needle; selected colonies were then expanded for DNA analysis. Clones were initially screened using diagnostic restriction digest or via PCR specific primers. Positive clones were subsequently confirmed using Sanger sequencing.

#### Western blot

SCN9A-HA cells were grown under described iPSC culture conditions. Cells were lysed with ice cold modified RIPA (Sigma) supplemented with protease inhibitor cocktail (cOmplete Mini, EDTA-free, Roche) and lysates were cleared by centrifugation. Total protein (10 μg) was incubated at 35°C for 5 min with 5X Lamelli Buffer (ThermoFisher). Proteins were separated on 4%–12% BIS-TRIS mini gels (ThermoFisher) and transferred to a nitrocellulose membrane using BIORAD Trans-blot wet transfer system. Membranes were blocked for 1 h in 8% skimmed milk, 0.1% PBS-T and incubated overnight with Anti-HA (1:1000, Cell Signaling Technology) and β-actin (1:10,000, Sigma) in blocking solution. Membranes were washed 3X in 0.1% PBS-T, then incubated at room temperature for 45 min with a HRP conjugated secondary antibody 1:10,000 (Amersham, G.E healthcare). Signal was detected with ECL Prime using chemiluminescent detection film (G.E. healthcare).

#### Schwann cell co-cultures

Schwann cell co-cultures were prepared as previously described (Clark et al., 2017). In brief, 30,000 rat Schwann cells were added to 30 DIV iPSC-derived nociceptors in Schwann cell basal medium [DMEM/F12 (ThermoFisher), 5 mg/ml insulin (Sigma), 100 mg/ml transferrin (Millipore), 25 ng/ml recombinant-human NGF (Peprotech), 25 ng/ml Selenium (Sigma), 25 ng/ml thyroxine (Sigma), 30 ng/ml progesterone (Sigma), 25 ng/ml triiodothyronine (Sigma) and 8 mg/ml putrescine (Sigma)]. Cells were either maintained in this medium where ‘non-myelinating’ conditions were required, or myelination was induced one week after Schwann cell addition by exposing the cells to myelination medium ((N2 medium, 1:300 phenol-free Matrigel (Corning), 5% charcoal-stripped FBS (ThermoFisher), 25 ng/ml recombinant- human NGF (Peprotech), 50mg/ml ascorbic acid (Sigma)). Myelinating co-cultures were maintained for a further 5 weeks before fixation for ICC analysis.

#### Immunocytochemistry

Cells were fixed in 4% paraformaldehyde (GIBCO) for 12 min and permeabilized with 0.1% Triton X-100 for 5 min. They were then blocked with 5% normal donkey or goat serum (Sigma) for 30 min and incubated with primary antibodies overnight at 4°C. The following primary antibodies were used: Mouse anti-HA 1:400 (Sigma), Rabbit anti-HA (1:800), Chicken anti-NF200 (1:10,000), Rat anti-MBP 1:400 (Abcam), Rabbit anti-Brn3a 1:500 (Millipore), Phalloidin-TRITC conjugate 1:150 (Sigma). Cells were washed 3X with 0.1% PBS-TX before the species appropriate Alexa Fluorophore secondary antibodies were applied (all 1:1000). Coverslips were mounted with VECTASHIELD Antifade Mounting Medium (Vector Laboratories). Immunostaining was visualized using a confocal microscope (Zeiss LSM 700) and images were acquired using the Zen Black software.

#### Neurite outgrowth assays

For long-term axonal outgrowth, neurites and cell bodies of mature iPSC-nociceptors were visualized by immunocytochemistry staining for NF200. 49 fields of view of a 12mm coverslip were acquired using GE IN Cell Analyzer 6000 build 6.1 at x10 objective. Neuronal cell bodies in mature cultures cluster, making accurate determination of cell number challenging; axonal coverage was therefore normalized to somal area (μm^2^) following confirmation of no significant difference between cell diameter of each group. Acquired images were imported into an IN Cell Developer Toolbox analysis software build 1.9.2 and analysis was performed automatically using a custom pipeline. In brief, a soma mask and total area network mask (neuronal somas and axons) were created from binary images. The soma mask was subtracted from the network to yield axonal area. Fields containing artifacts e.g., tears in axons or large debris, were manually excluded by eye. To compare fields of similar neuronal density, fields with a soma area between 1.5x10^4^ μm^2^ and 4.5x10^4^ μm^2^ (equivalent to 8.5%–25% area) were used for analysis. A minimum of 9 coverslips from each group were analyzed.

For outgrowth following dissociation, mature iPSC-nociceptors were enzymatically treated with 30 min 0.1% TrypleE (ThermoFisher), followed by mechanical dissociation with a fire polished glass pipette. Single cells were re-plated onto matrigel treated coverslips at low density before fixation, immunocytochemistry, and analysis 12 h later. The proportion of neurons with none, short (longest neurite < 3x soma diameter) or long neurites (longest neurite > 3x soma diameter) were scored manually. For neurite length, images were acquired and neurites were semi-automatically traced and measured using the ImageJ plugin- Simple Neurite Tracer (Longair et al., 2011). All experiments were performed by an experimenter blind to groups.

#### RNA extraction and cDNA synthesis

Total RNA extraction was performed using Tripure and High Pure RNA Isolation Kit (Roche). An on-column DNase digestion step was included to eliminate contaminating gDNA. RNA was eluted in nuclease free ddh_2_0. Synthesis of cDNA was performed using EvoScript Universal cDNA Master (Roche).

#### RT-qPCR

RT-qPCR was performed using SYBRgreen qPCR master mix (Roche) according to the manufacturer’s instructions. Samples were prepared in triplicate in 384-well reaction plates then run on LC480 II System (Roche). Primers were designed using Primer-BLAST (https://www.ncbi.nlm.nih.gov/tools/primer-blast/). Primer efficiency and specificity were validated before experimental use. Gene expression for each target primer was normalized against the reference gene YWHAZ using the ΔΔCT method.

#### RNA-sequencing

Analysis of sequencing data was processed in a workflow similar to previously (Baskozos et al., 2019). Publicly available RNA-seq data were mapped in GRC.h.38 Human Genome using the STAR aligner with the ENCODE standard options. Read counts were calculated at the gene level using HTSeq and the ENSEMBL gene set annotation GRC.h.38.88. Raw counts were normalized using the effective library size and transformed using the variance stabilizing transformation (VST) in R using DESeq2. Principal component analysis (PCA) plots were generated from 100 randomly selected samples per GTEx human tissue, the human iPSC and iPSC-nociceptor, the single-cell Human iPSC and iPSC-nociceptor and the human DRG RNA-seq data.

The top 500 ENSEMBL genes ranked by the standard deviation of their VST counts were selected for PCA. Data were centered and scaled before PCA and samples are projected to standardized components. Ellipses represent the 95% CI of a tissue’s gene expression distribution. Distances between samples in the plot are proportional to Mahalanobis distance, i.e standard deviations from a distribution’s mean.

#### Electrophysiology of HEK293T cells

Human embryonic kidney HEK293T cells were grown in a Dulbecco’s modified Eagle’s culture medium (DMEM/F-12, Invitrogen) containing 10% fetal bovine serum and maintained under standard conditions at 37°C in a humidified atmosphere containing 5% CO2. Cells were transfected using the jetPEI transfection reagent (Polyplus-transfection Inc.) with either wild-type or mutant Na_V_1.7 channel combined with β1 and β2 subunits (40:1 ratio). Recordings were made 48 to 72 h after transfection.

Whole-cell voltage clamp experiments were performed on transfected HEK293T cells exhibiting both red and green fluorescence in the expectation that such cells would also express Na_V_1.7, combined with β1 and β2 subunits. All the recordings were conducted at room temperature using an Axopatch 200B Amplifier, the Digidata 1550B Low Noise Data Acquisition System and the pClamp10.6 software (Molecular Devices). Data were filtered at 5kHz and digitized at 20kHz. Capacity transients were cancelled and series resistance compensated at 70%–90% in all experiments. The extracellular solutions contained (in mM): 140 NaCl, 3 KCl, 1 CaCl_2_, 1 MgCl_2_, 10 HEPES, pH 7.3 with NaOH (adjusted to 320 mOsm/L with glucose). Patch pipettes were filled with an internal solution containing (in mM) 140 CsF, 10 NaCl, 1 EGTA, 10 HEPES, pH 7.3 with CsOH (adjusted to 310 mOsm/L with glucose) and had a typical resistance of 2-3MΩ.

#### Electrophysiology of iPSC nociceptors

iPSC nociceptors were assessed for their biophysical properties 50-70 days following addition of growth factors. At this time point, neurons exhibit mature electrophysiological properties (Weir et al., 2017), gene expression changes have plateaued (Young et al., 2014), and high levels of Na_V_1.7 are expressed at the membrane (Figure 5E) Whole-cell patch clamp recordings using an Axopatch 200B amplifier and Digidata 1550 acquisition system (Molecular Devices) were performed at room temperature (22°C). Data were low-pass filtered at 2 kHz and sampled at 10 kHz. Series resistance was compensated 60%–80% to reduce voltage errors. All data were analyzed by Clampfit 10 software (Molecular Devices)

##### Current clamp

Filamental borosilicate glass capillaries (1.5 mm OD, 0.84 mm ID; World Precision Instruments) were pulled to form patch pipettes of 3–5 MΩ tip resistance and filled with internal solution containing (mM): 130 KCl, 1 MgCl_2_, 5 MgATP, 10 HEPES, and 0.5 EGTA; pH was adjusted to 7.3 with KOH and osmolarity set to 305 mOsm. Extracellular solution was perfused at a continuous rate of 1ml/min and contained (mM): 140 NaCl, 3 KCl, 2 MgCl_2_, 2 CaCl_2_, 10 HEPES and 10 glucose; pH was adjusted to 7.3 with NaOH and osmolarity was set to 315 mOsm. Resting membrane potential was assessed in bridge mode, while firing properties were assessed in current clamp mode. Input resistance (R_Input_) was derived from the membrane potential deflection caused by a 20 pA hyperpolarising current pulse at −60 mV. Cells were depolarised from a holding potential of −60 mV by current steps (50 ms) of increasing magnitude (Δ10 pA) until an action potential was generated, to determine rheobase. Repetitive firing was evoked by prolonged (500ms) depolarising steps of increasing increments (Δ25 pA). Depolarisation current ramps of 1nA amplitude were given over 100-1000ms to assess firing in response to slow depolarisation. The ability of neurons to fire at high frequency was assessed by seventeen current stimuli of 1.5x rheobase (50ms) given at 20Hz.

##### Voltage clamp

To assess voltage-gated Na^+^ currents, patch pipettes of 1.5-3 MΩ were filled with an internal solution containing (mM): 140 CsF, 10 NaCl, 1 EGTA and 10 HEPES; pH was adjusted to 7.3 with CsOH and osmolarity set to 305 mOsm. The extracellular solution contained (mM): 70 NaCl, 50 N-methyl-d-glucamine, 20 Tetraethylammonium chloride, 1 CaCl_2_, 3 KCl, 1 MgCl_2_, 10 HEPES, 10 Glucose and 0.1 CdCl_2_; pH was adjusted to 7.3 with NaOH and osmolarity set to 305 mOsm. To assess voltage-gated Na^+^ currents, membrane potential was stepped from −80mV to +40mV in 10mV increments, from a holding potential of −100 mV. Intersweep intervals were 10 s. To mitigate inconsistencies associated with space clamp and voltage errors, the Na^+^ current was calculated at the potential, which gave the peak inward current. Recordings were discarded if series resistance > 10 MΩ or deviated by > 20% during the recording. Linear leak subtraction was performed using P/4 leak subtraction.

#### Drugs

All solution chemicals were purchased from Sigma-Aldrich. BIIB074 (Axon Medchem) and PF-05089771 (Sigma Aldrich) were dissolved in DMSO at 100mM and 1mM, respectively, aliquoted and stored at −20°C until the day of use. Cells were bathed in drug or vehicle for at least 30 min prior to recording.

### Quantification and Statistical Analysis

Data are shown as the mean ± SEM, unless otherwise stated. A Student’s t test was used to compare the mean of two groups and when data were not normally distributed a non-parametric test was applied (Mann-Whitney). A one-way ANOVA was used when more than two groups existed. For patch clamp experiments, two-way ANOVA with Holm-Sidak post hoc analysis was used to assess firing in response to prolonged suprathreshold and graded stimuli. Comparison of rheobase and firing to high-frequency stimulation was tested by Kruskal-Wallis followed by post hoc Dunn’s test. Current densities recorded in heterologous expression systems were compared by one-way ANOVA followed by post hoc Dunn’s test. Verbal pain intensity ratings and NPS responses measured by fMRI were compared using a Mann-Whitney U test. Student’s unpaired t test was used to compare *SCN9A* mRNA levels. Sample sizes are detailed in each figure legend. Significance for all experiments was placed at p < 0.05. Statistical tests were carried out with GraphPad prism or SigmaStat.

## Supplemental Information

Document S1. Figures S1–S7 and Tables S1–S4

Document S2. Article plus Supplemental Information

Video Abstract
