# Supplementary material for: Defining the Functional Role of NaV1.7 in Human Nociception
Source: Neuron. 2019 Mar 6;101(5):905–919.e8. doi: 10.1016/j.neuron.2019.01.047 (PMC6424805; doi:10.1016/j.neuron.2019.01.047)
Supplement: Document S1. Figures S1–S7 and Tables S1–S4 [file mmc1.pdf]

Neuron, Volume *101*

Supplemental Information

Defining the Functional Role of $Na_V1.7$ in Human Nociception

Lucy A. McDermott, Greg A. Weir, Andreas C. Themistocleous, Andrew R. Segerdahl, Iulia Blesneac, Georgios Baskozos, Alex J. Clark, Val Millar, Liam J. Peck, Daniel Ebner, Irene Tracey, Jordi Serra, and David L. Bennett

Supplementary Information- Defining the functional role of $Na_V1.7$ in human nociception

Table S1- related to Figure 1. *SCN9A* mutations of CIP patients

Identifier	Mutation, reference transcript NM_002977.3	Mutation functionally characterised
**CIP 1**	Compound heterozygote*:	
	c.2488C>T (R830X) - premature stop codon in coding exon 16	No
	c.5318delA (FS1773) - in exon 27, 1bp deletion that induces a frameshift at position 1773 in the C terminal domain of the channel.	No
**CIP 2**	Compound heterozygote:	
	c.377+5C>T – intronic variant	No
	c.2686C>T (R896W) – in exon 16, amino acid changing, affects a conserved region of the protein, and is predicted to cause an alteration in the ion transport region of the protein	No
**CIP 3**	Compound heterozygote:	
	c.2691G>A (Y897X) – premature stop codon	Yes (Cox et al. 2006)
	c.5173G>C (G1725R) – in exon 27, amino acid changing, affects a highly conserved region of the protein and is predicted to cause major alteration in the sixth transmembrane region of the protein	No

*Mutations were previously reported, but not functionally characterised (Ramirez et al. 2014).

Table S2- related to Figure 1: Summary of the relevant nerve conduction studies for three study participants.

CIP 1			
	Left	Right	Normative reference range
Sensory nerve action potentials			
Median nerve onset latency	2.7 ms	2.5 ms	≤ 3.2 ms
Median nerve amplitude (peak to peak)	14.5 µV	11.4 µV	≥ 13.0 µV
Median nerve conduction velocity	69.3 m/s	60.5 m/s	
Ulnar nerve onset latency	2.3 ms	2.2 ms	≤ 2.9 ms
Ulnar nerve amplitude (peak to peak)	8.5 µV	10.2 µV	≥ 8 µV
Ulnar nerve conduction velocity	55.5 m/s	60.5 m/s	
Sural nerve onset latency	2.7 ms	2.7 ms	≤ 3.5 ms
Sural nerve amplitude (peak to peak)	4.6 µV	4.1 µV	≥ 5 µV

Sural nerve conduction velocity	44.9 m/s	51.2 m/s	
**Motor compound action potentials**			
Median nerve onset latency	3.4 ms	3.3 ms	≤ 4.6 ms
Median nerve amplitude (baseline to peak)	5.2 mV	6.4 mV	≥ 4.8 mV
Median nerve conduction velocity	61.4 m/s	58.3 m/s	≥ 50.2 m/s
Common peroneal nerve onset latency	5.1 ms	4.0 ms	≤ 6.4 ms
Common peroneal nerve amplitude (baseline to peak)	3.4 mV	2.8 mV	≥ 2.6 mV
Common peroneal nerve conduction velocity	50.3 m/s	53.0 m/s	≥ 38.2 m/s
**CIP 2**			
	**Left**	**Right**	**Normative reference range**
**Sensory nerve action potentials**			
Median nerve onset latency	2.2 ms	2.2 ms	≤ 3.2 ms
Median nerve amplitude (peak to peak)	9.5 µV	22.6 µV	≥ 13.0 µV
Median nerve conduction velocity	67.0 m/s	60.1 m/s	
Ulnar nerve onset latency	2.3 ms		≤ 2.99 ms
Ulnar nerve amplitude (peak to peak)	8.1 µV		≥ 8 µV
Ulnar nerve conduction velocity	53.4 m/s		
Sural nerve onset latency		2.4 ms	≤ 3.5 ms
Sural nerve amplitude (peak to peak)		5.1 µV	≥ 5 µV
Sural nerve conduction velocity		56.9m/s	
**Motor compound action potentials**			
Median nerve onset latency	3.0 ms	3.3 ms	≤ 4.6 ms
Median nerve amplitude (baseline to peak)	5.8 mV	5.4 mV	≥ 4.8 mV
Median nerve conduction velocity	63.0 m/s	57.8 m/s	≥ 50.2 m/s
Common peroneal nerve onset latency		3.9 ms	≤ 6.4 ms
Common peroneal nerve amplitude (baseline to peak)		2.9 mV	≥ 2.6 mV
Common peroneal nerve conduction velocity		45.2 m/s	≥ 38.2 m/s
**CIP 3**			
	**Left**	**Right**	**Normative reference range**
**Sensory nerve action potentials**			
Median nerve onset latency	2.5	2.3	≤ 3.2 ms
Median nerve amplitude (peak to peak)	31.4 µV	24.7 µV	≥ 13.0 µV
Median nerve conduction velocity	57.1 m/s	55.5 m/s	
Sural nerve onset latency	2.0	1.9	≤ 3.5 ms
Sural nerve amplitude (peak to peak)	7.5 µV	9.6 µV	≥ 5.0 µV
Sural nerve conduction velocity	55.3 m/s	53.0 m/s	
**Motor compound action potentials**			
Median nerve onset latency	2.7 ms	2.9 ms	≤ 4.3 ms
Median nerve amplitude (baseline to peak)	7.7 mV	8.4 mV	≥ 4.3 mV
Median nerve conduction velocity	51.5 m/s	53.4 m/s	≥ 47.2 m/s
Common peroneal nerve onset latency	3.3 ms		≤ 6.4 ms
Common peroneal nerve amplitude (baseline to peak)	6.5 mV		≥ 1.1 mV
Common peroneal nerve conduction velocity	51.2 m/s		≥ 37.2 m/s

The nerve recordings highlighted in yellow were not suitable for neurophysiology study. This was due to past injures and subsequent distortion of the anatomy, The normative ranges are from (Buschbacher & Orahlow 2006)

Table S3- related to Figure 3: Conduction velocity and ADS for the main C-fibre types.

	Type 2	Type 3	Type 4
**CV (m/s**			
CIP	0.91±0.39 (10)	0.86±0.40 (5)	0.59±0.25 (23)
Normative data	0.72 ± 0.35 (16)	0.82 ± 0.59 (5)	0.63±0.19 (41)
Significance	n.s	n.s	n.s
**ADS 2Hz (%)**			
CIP	4.76±1.37 (10)	1.26±0.43 (5)	4.38±2.84 (23)
Normative data	5.2 ± 1.6 (14)	0.8 ± 0.5(5)	4.90±2.20 (41)
Significance	n.s	n.s	n.s

Conduction velocity (CV) and percentage of ADS of conduction velocity after 3 min at 2Hz (ADS 2Hz). Data is pooled from all patient recordings (n=38) and represents mean ± SD (n). Student's *t*-test was used to compare values to normative data, which was taken from (Serra et al. 1999; Campero et al. 2004). Note, Type 1 fibres (C-nociceptors) were absent from all recordings.

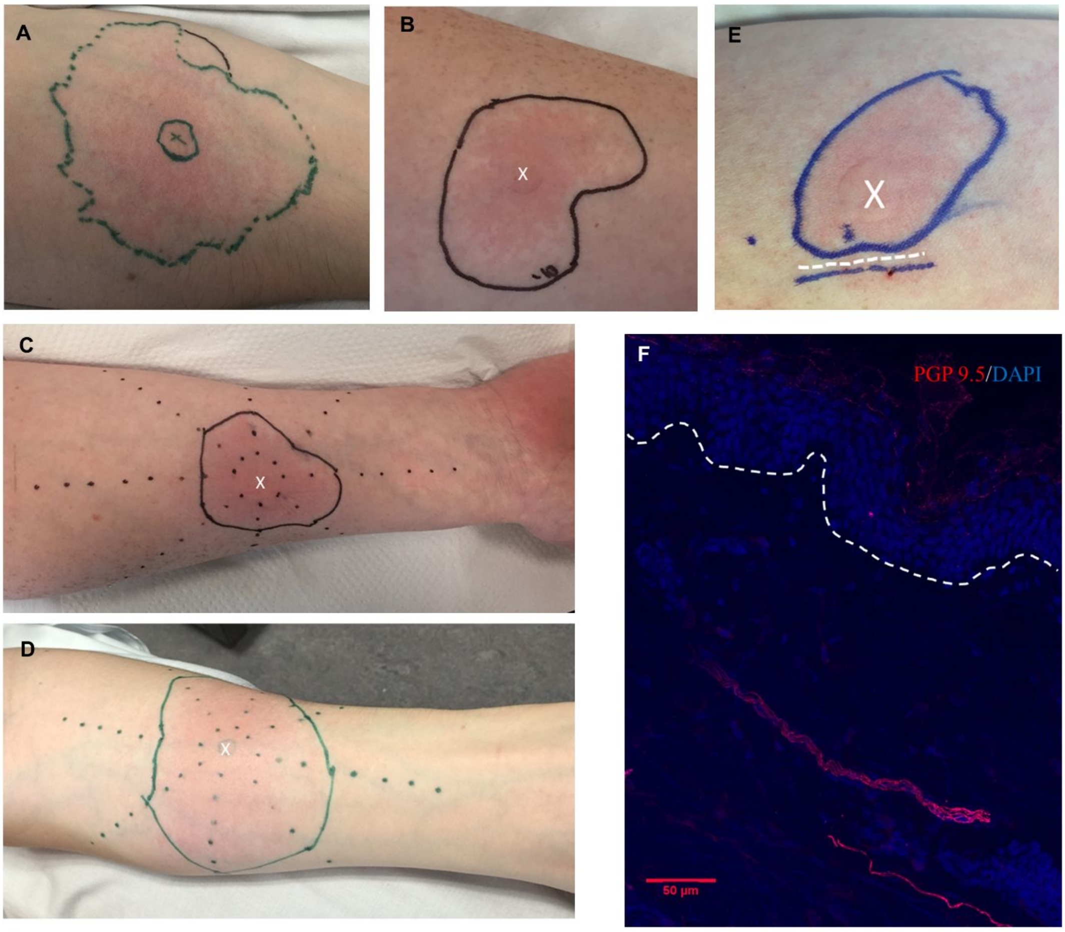

Figure S1- related to Figure 1. Flare response to histamine and mustard oil.

A) & B) Flare responses 10 minutes after 2% histamine iontophoresis into the skin over the forearm of the two other study participants with CIP. “X” marks the area of ionotophoresis and the coloured circles the extent of the flare. The participants did not report pruritus. C) & D) Flare response 10 minutes after topical application of 30% mustard oil over the forearm of the two other study participants with CIP. “X” marks the area of mustard oil application and the green circle the extent of the flare response. The participant did not report any spontaneous pain or discomfort. E) Pre-emptive injection of 2ml of 1% lignocaine (local anaesthetic) blocked the histamine flare. Dashed lines mark the area of lignocaine infiltration and “X” marks the area of iontophoresis. The flare did not extend beyond the area of lignocaine infiltration. F) Skin biopsy taken from the proximal thigh of a study participant with congenital insensitivity to pain demonstrating the absence of intra-epidermal nerve fibres and the presence of dermal fibres. The dashed line represents the division between the epidermis and dermis.

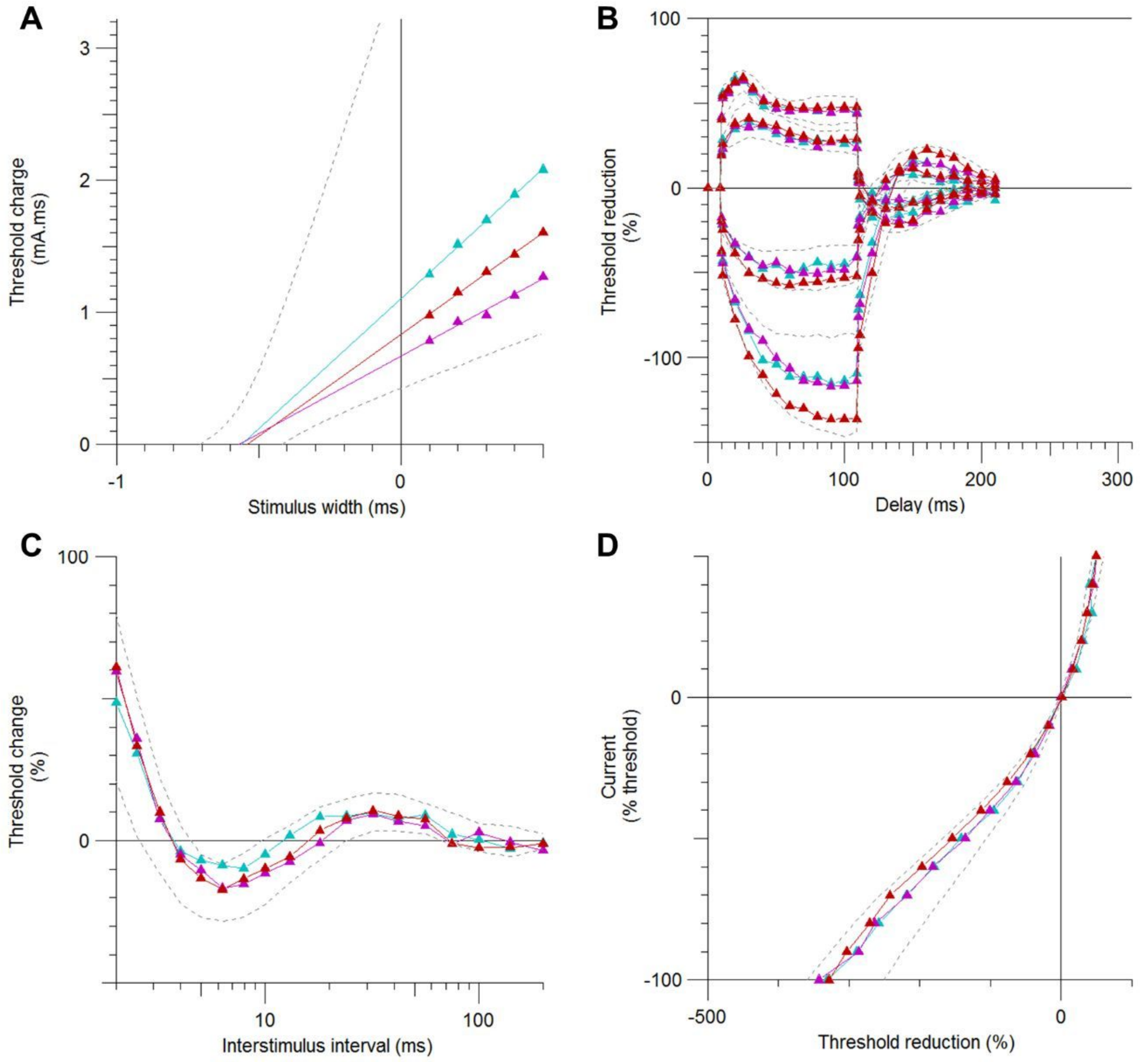

Figure S2- related to Figure 1. Sensory nerve excitability.

Measurements were performed using automated QTRAC software. Sensory nerve action potentials were recorded from the index finger using surface electrodes after stimulation of the median nerve at the wrist. Each nerve excitability recording consists of four discrete tests that include assessment of A) stimulus–duration time constant, B) threshold electrotonus, C) the current–threshold relationship, D) the recovery cycle. All recordings from the study participants fell within the 95% CI for healthy individuals (represented by the dashed lines).

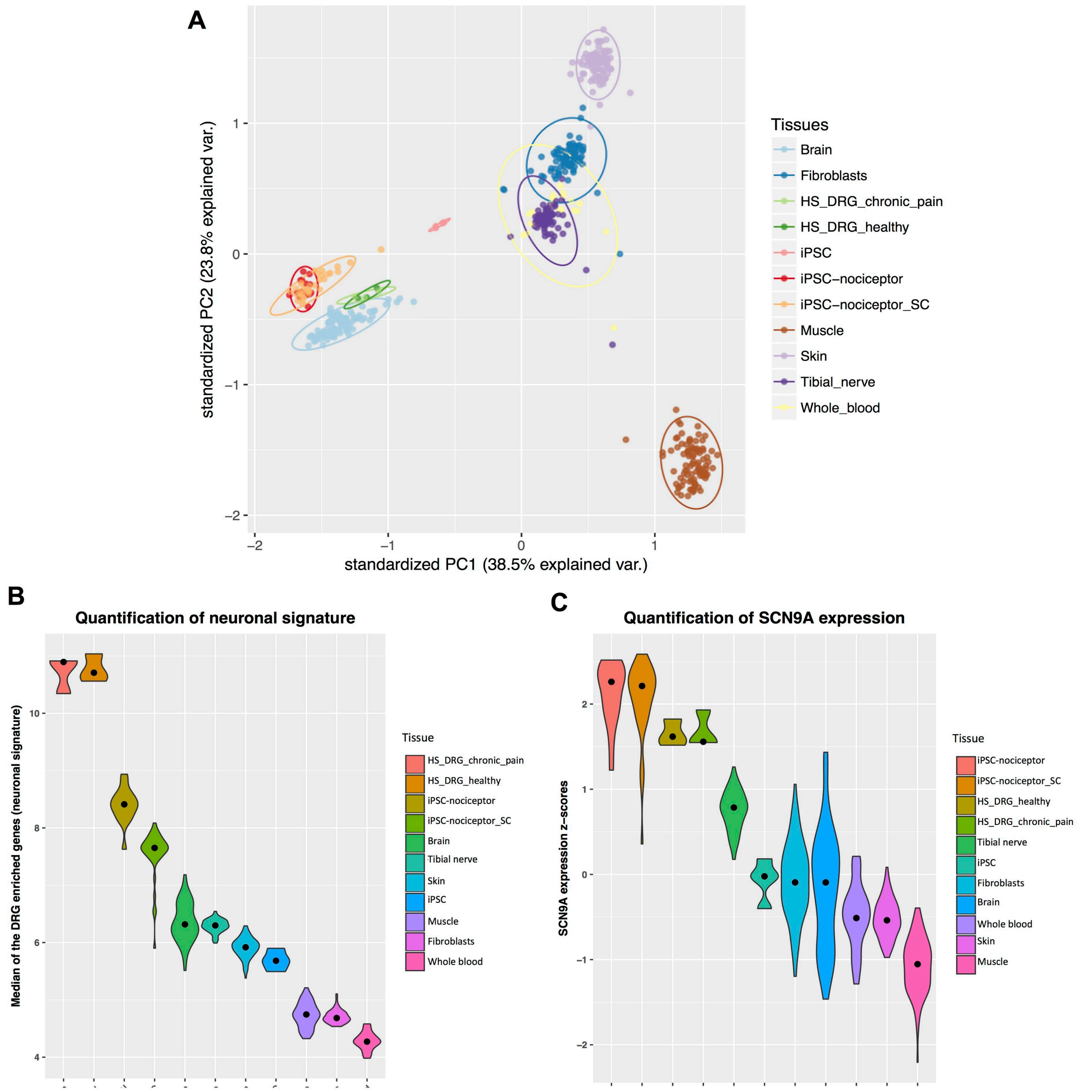

Figure S3- related to Figure 5. RNA-seq analysis of iPSC-nociceptors.

A) iPSC nociceptors derived in this study (iPSC-nociceptors) and previously published single-cell data (Schwartzentruber et al. 2018) (iPSC-nociceptor_SC), iPSC, human DRG from healthy patients (HS_DRG_healthy) and chronic pain patients (HS_DRG_chronic_pain)(Ray et al. 2018) and other GTEx human tissues projected onto the two first standardised principal components defined from RNA-seq gene expression counts. Ellipses represent the 95% confidence interval of a tissue's gene expression distribution. Distances between samples in the plot are proportional to their Mahalanobis distance.

B) Violin plots visualising the kernel density estimation of the average expression of human DRG enriched genes defined in Ray et al. 2018. Black dot represents the median.

C) Violin plots showing the z-score transformed expression of the SCN9A gene across tissues. Black dot represents the median. Gene counts for all plots were transformed using the variance stabilising transformation.

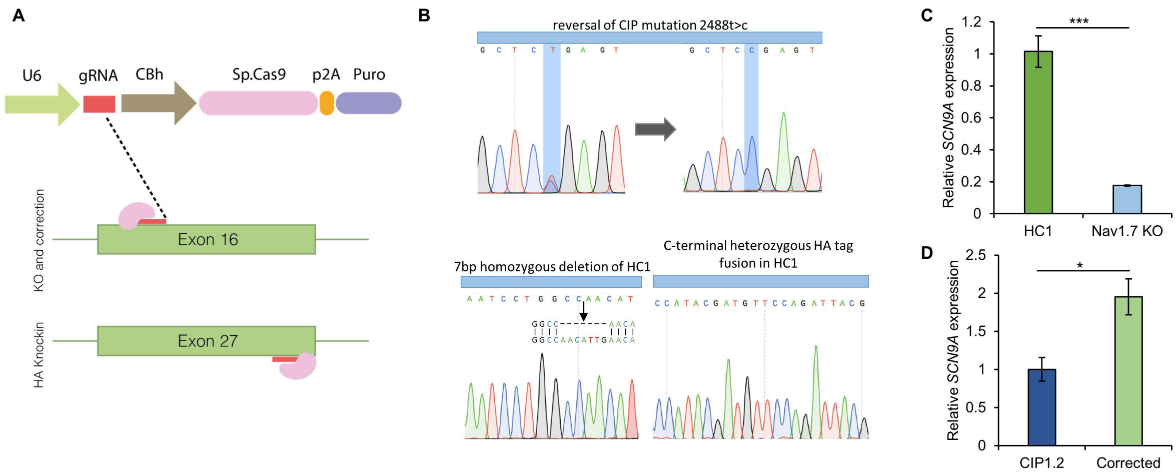

Figure S4- related to Figures 5 and 6. CRISPR-mediated genome engineering of the *SCN9A* locus and protein localisation.

A) SCN9A targeting strategy. Wild type S.p.Cas9 was transiently expressed in iPSC using plasmid based transfection (Addgene PX459 V.2). For each target the guides were inserted between BbsI cut sites. Where a repair template was required, a long single stranded oligo donor template was transfected simultaneously with the S.p.Cas9 vector. Chromatograms depict successfully targeted and modified loci.

B) Chromatograms for genome-edited lines. Panels illustrate successful correction of 2488t>c to the wild-type sequence (corrected) in the patient line CIP1.2. Below, a 7bp deletion leading to premature stop codon R842X (Na_V1.7 KO) and C-terminal in frame insertion of the haemagglutinin epitope tag (YPYDVPDYA) (*SCN9A*-HA).

C) Relative expression of *SCN9A* mRNA in control (HC1) compared to Na_V1.7 KO. Expression was normalised to the housekeeping gene YWHAZ. Data represents mean ± s.e.m of four independent differentiations. *SCN9A* mRNA expression was significantly reduced in CRISPR generated knockout, approximately 5.6 fold reduced. This is consistent with non-sense mediated decay of the targeted transcript. ***p<0.001, Student's t-test.

D) Relative expression of *SCN9A* mRNA in CIP1.2 compared to CRISPR/Cas9 corrected clone (Corrected). Expression was normalised to the housekeeping gene YWHAZ. Data represents mean ±

s.e.m of three independent differentiations. *SCN9A* mRNA expression was rescued by allelic correction of the R830X mutation. *p<0.05, Student's *t*-test.

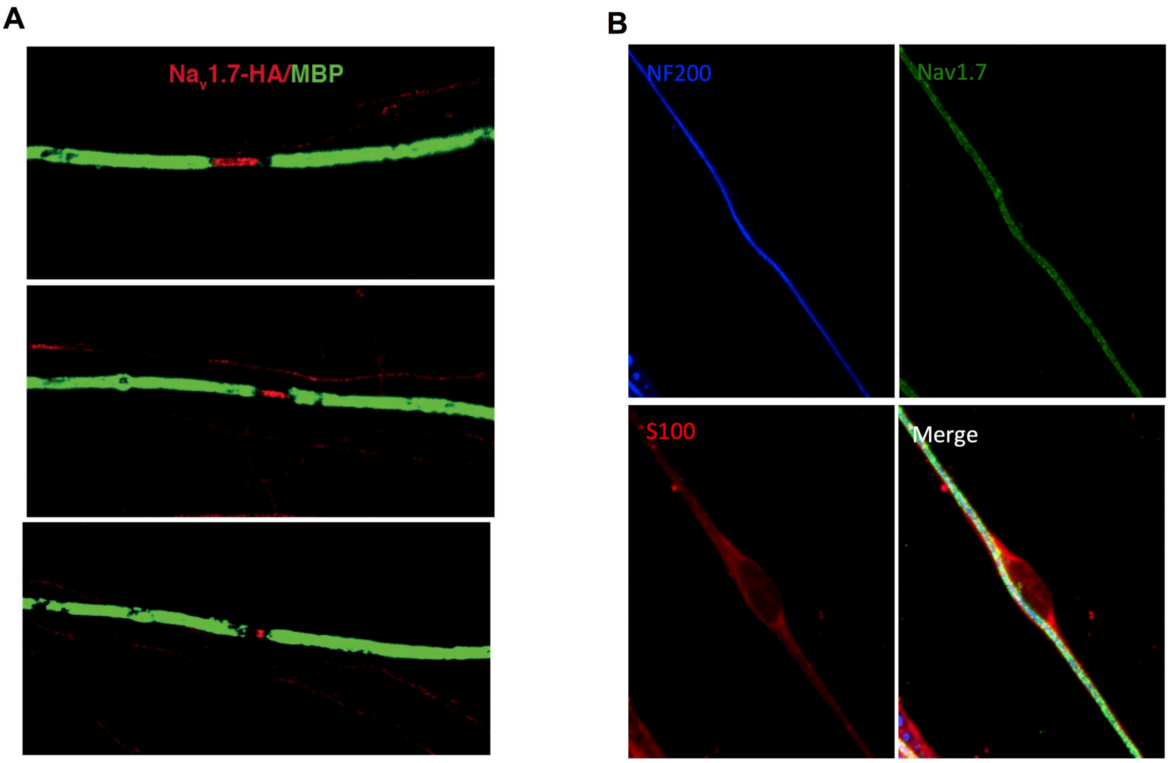

Figure S5- related to Figure 5. iPSC-nociceptor schwann cell co-culture.

A) *In vitro* maturation of myelinating iPSC- nociceptor co-cultures. Maturation (bottom to top) of nodes of Ranvier causes lateral compaction of Na_V1.7 expression. Myelin basic protein (green), Na_V1.7 -HA (red).

B) Representative image of Na_V1.7 expression in an iPSC-nociceptor when a Schwann cell is aligned, but not myelinating the neurite. NF200 (neurite- blue), Na_V1.7 (green) and S100 (Schwann cell- red).

Table S4- related to Figure 6. Biophysical properties of iPSC lines studied.

	HC1	HC3	HC2	cCIP 1.1	cCIP 1.2	cCIP 2	Corrected	Na_V1.7 KO
**RMP (mV)**	-61.9 ±0.8	-60.7 ±1.2	-58.5 ±1.1	-63.1 ±1.1	-61.4 ±0.96	-62.7 ±1.5	-58.2 ±0.8	61.1 ±0.9
**R_{Input} (MΩ)**	213.3 ±9.4	198.3 ±17.0	205.0 ±17.3	210.9 ±11.4	211.7 ±15.8	176.0 ±12.3	208.5 ±11.3	181.1 ±8.4
**Cap (pF)**	32.5 ±1.4	34.9 ±4.2	34.5 ±3.1	28.2 ±1.9	36.1 ±2.2	36.2 ±1.4	39.4 ±2.2	36.1 ±1.3

**No. of cells**	90	22	26	32	48	29	34	53
**RIn<100 MΩ, excluded**	0 (0%)	1 (4.5%)	2 (7.7%)	2 (6.3%)	5 (10.4%)	3 (10.3%)	0 (0%)	3 (5.7%)
**No shoulder, excluded**	10 (11%)	2 (9.1%)	4 (15.4%)	3 (9.4%)	7 (14.6%)	4 (13.8%)	1 (3%)	5 (9.4%)

No statistical differences were found between any lines and control (HC1). *p*>0.05, Kruskal Wallis followed by Dunn's multiple comparison test. Cap- capacitance, R_{In}- input resistance

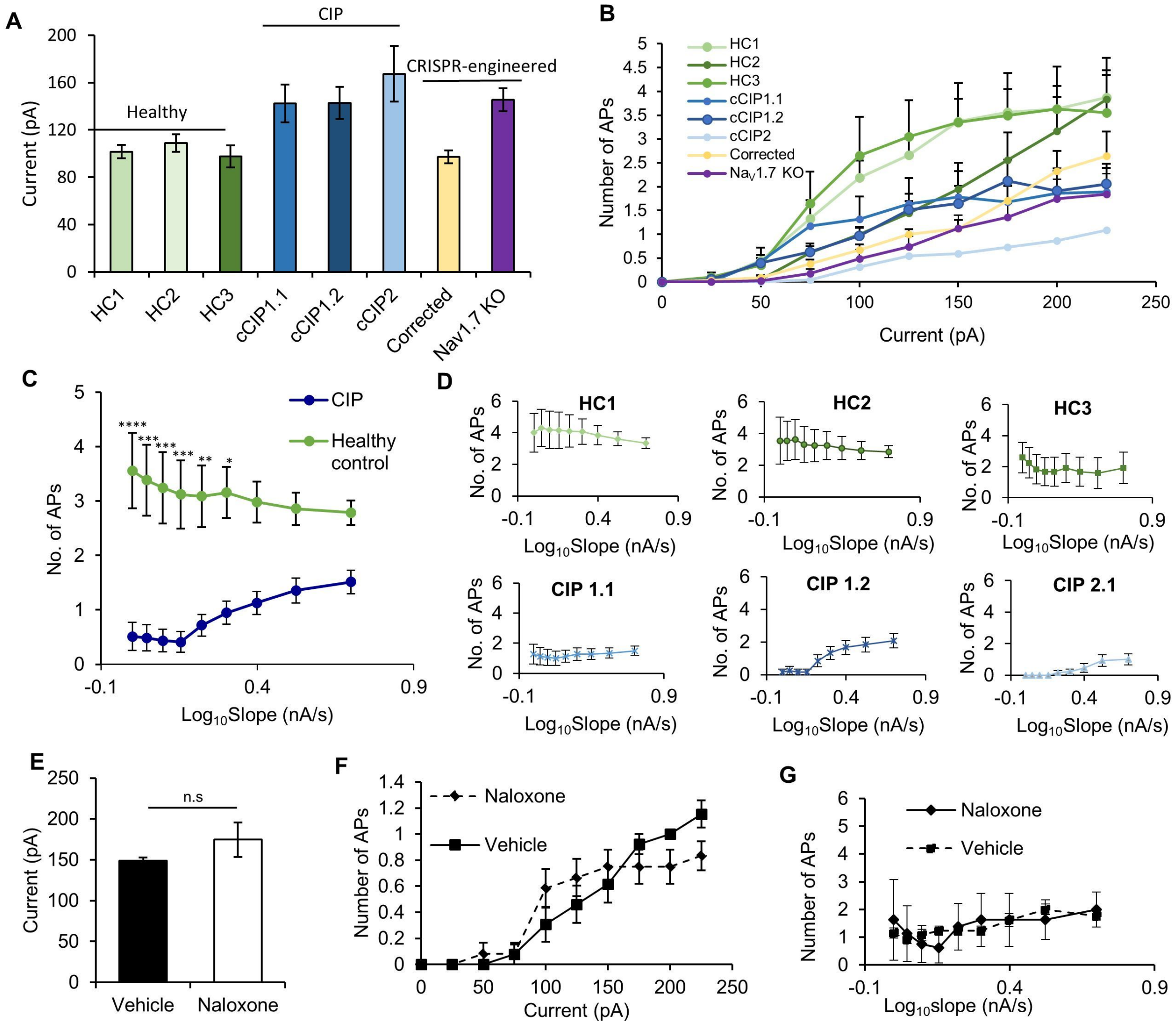

Figure S6- related to Figure 6. Excitability of individual iPSC lines and effect of naloxone.

A) Minimum current required to generate an action potential in individual iPSC lines. Derived from incremental current injections (Δ10pA) of 50ms duration. Sample sizes: HC1- 80 cells, HC2- 20 cells,

HC3- 19 cells, cCIP1.1- 27 cells, cCIP1.2- 36 cells, cCIP2- 22 cells, Corrected- 33 cells and $Na_V1.7$ KO- 45 cells.

B) Firing in response to incremental (Δ25pA) and prolonged (500ms) current injection of individual iPSC lines. Same sample sizes as A). All data represents mean ± s.e.m of at least three independent differentiations.

C) Firing in response to 1nA current injection given over 100-1000ms in 100ms increments (as per Figure 6). $^{*}p<0.05$, $^{**}p<0.01$, $^{**}p<0.001$, $^{****}p<0.0001$, Two-way ANOVA followed by post-hoc Sidak's multiple comparison test. All data represents mean ± s.e.m from pooled data of Healthy control (HC1- 31 cells, HC2- 13 cells, HC3- 13 cells) and CIP (CIP1.1- 14 cells, CIP1.2- 12 cells, CIP2.1- 13 cells) lines.

D) Data from *C* plotted for each individual cell line.

E) Minimum current required to fire an action potential of $Na_V1.7$ KO neurons treated >30 minutes with Vehicle (0.01% DMSO) (n=14 cells) or 1µM Naloxone (n=12 cells). N.s p>0.05, Student's unpaired *t*-test.

F) Firing of treated neurons in response to incremental (Δ25pA) and prolonged (500ms) current injections. No differences observed between the groups, two-way ANOVA followed by post-hoc Sidak's multiple comparison test.

G) Firing of treated neurons in response to 1nA current given over different periods of time. No differences observed between the groups, two-way ANOVA followed by post-hoc Sidak's multiple comparison test.

All data represents mean ± s.e.m.

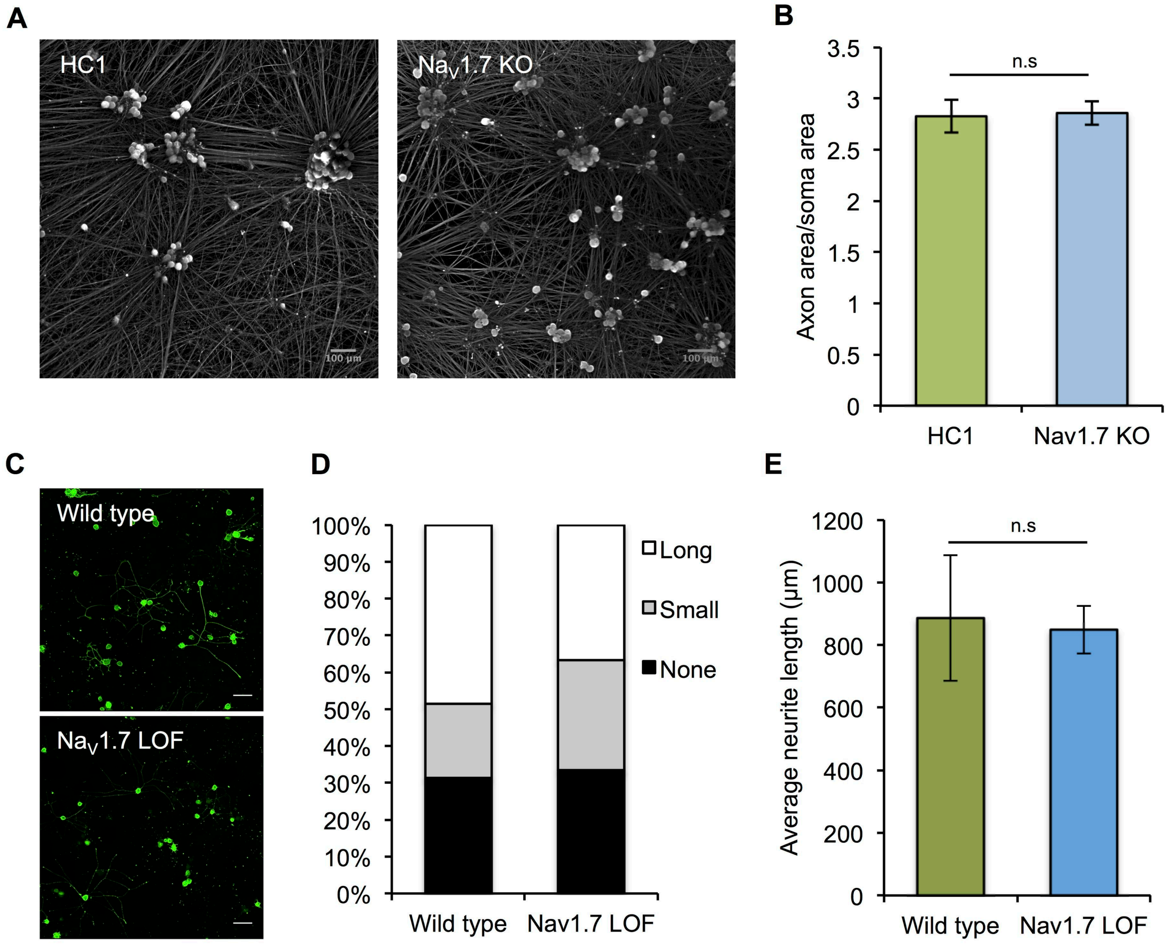

Figure S7- related to Figure 6. $Na_V1.7$ is not critical for neurite outgrowth in vitro

A) Representative images of DIV 60 HC1 and $Na_V1.7$ KO iPSC-nociceptors infected 2 weeks previously with pAAV-CAMKII GFP, demonstrating extensive neurite projections.

B) Quantification of axon area relative to somal area. Data represents the mean ± s.e.m of 11 (HC1) and 9 ($Na_V1.7$ KO) coverslips taken from three independent differentiations.

C) iPSC-nociceptors dissociated and re-plated at DIV 60. Representative images of neurons 12 hours post re-plating processed by immunocytochemistry for NF200 (green) staining

D) Quantification of the proportion of neurons with none, small (at least one neurite <3x soma diameter) or long (at least one neurite >3x soma diameter) neurite projections. Wild type group represents pooled data from HC1, HC2 and HC3 healthy control lines (n=592 neurons). $Na_V1.7$ LOF data represents pooled data from CIP lines (CIP1.1, CIP1.2 and CIP2.1) and $Na_V1.7$ KO line (n=845 neurons). In all cases, data is derived from at least three independent experiments.

E) Quantification of neurite length per neuron. Wild type group represents mean ± s.e.m of HC1, HC2 and HC3 healthy control lines (n=3, total- 134 neurons). $Na_V1.7$ LOF data represents mean ± s.e.m of

CIP lines (CIP1.1, CIP1.2 and CIP2.1) and $Na_V1.7$ KO line (n=4, total- 205 neurons). In all cases, data is derived from at least three independent experiments. N.s $p>0.05$, Student's unpaired *t*-test.
